# AXL/CDCP1/SRC axis confers acquired resistance to osimertinib in lung cancer

**DOI:** 10.1038/s41598-022-12995-8

**Published:** 2022-05-28

**Authors:** Yuichi Murakami, Daiki Kusakabe, Kosuke Watari, Akihiko Kawahara, Koichi Azuma, Jun Akiba, Masahiko Taniguchi, Michihiko Kuwano, Mayumi Ono

**Affiliations:** 1 Cancer Translational Research Center, St. Mary’s Institute of Health Sciences, Kurume, Fukuoka Japan; 2 Department of Pharmaceutics, Graduate School of Pharmaceutical Sciences, Kyushu University, Fukuoka, Japan; 3 Physical Chemistry for Life Science Laboratory, Faculty of Pharmaceutical Sciences, Kyushu University, Fukuoka, Japan; 4 Department of Pharmacology, School of Medicine, University of California, San Diego, La Jolla, CA USA; 5 Department of Diagnostic Pathology, Kurume University Hospital, Kurume, Fukuoka Japan; 6 Division of Respirology, Neurology and Rheumatology, Department of Internal Medicine, Kurume University School of Medicine, Kurume, Fukuoka Japan; 7 Department of Surgery, St. Mary’s Hospital, Kurume, Fukuoka Japan; 8 Department of Pharmaceutical Oncology, Graduate School of Nursing, St. Mary’s College, 422 Tsubukuhonmachi, Kurume, Fukuoka 830-8558 Japan

**Keywords:** Non-small-cell lung cancer, Oncogenes

## Abstract

Osimertinib, a third-generation EGFR-TKI, has nowadays been applied to non-small cell lung cancer harboring activated EGFR mutation with or without T790M, but ultimately develop resistance to this drug. Here we report a novel mechanism of acquired resistance to osimertinib and the reversal of which could improve the clinical outcomes. In osimertinib-resistant lung cancer cell lines harboring T790M mutation that we established, expression of multiple EGFR family proteins and MET was markedly reduced, whereas expression of AXL, CDCP1 and SRC was augmented along with activation of AKT. Surprisingly, AXL or CDCP1 expression was induced by osimertinib in a time-dependent manner up to 3 months. Silencing of CDCP1 or AXL restored the sensitivity to osimertinib with reduced activation of SRC and AKT. Furthermore, silencing of both CDCP1 and AXL increased the sensitivity to osimertinib. Either silencing of SRC or dasatinib, a SRC family kinase (SFK) inhibitor, suppressed AKT phosphorylation and cell growth. Increased expression of AXL and CDCP1 was observed in refractory tumor samples from patients with lung cancer treated with osimertinib. Together, this study suggests that AXL/SFK/AKT and CDCP1/SFK/AKT signaling pathways play some roles in acquired osimertinib resistance of non-small cell lung cancer.

## Introduction

Treatment with first and second generation epidermal growth factor receptor-tyrosine kinase inhibitors (EGFR-TKIs), such as gefitinib, erlotinib, and afatinib, has contributed to therapeutic responses of patients with lung cancer and treatment-naïve oncogenic and activated mutant EGFR (mutEGFR)^1–3^. However, most patients ultimately develop acquired resistance to EGFR-TKIs and approximately 60% of such recurrent patients harbor a secondary resistant EGFR mutation involving T790M^4,5^. Osimertinib has been further developed by selective targeting of mutEGFR T790M, and is highly effective in patients with T790M-mediated resistant tumors^6,7^. Osimertinib demonstrates robust objective response rates and prolonged progression-free survival in treatment-naïve mutEGFR patients with advanced non-small cell lung cancer **(**NSCLC) as a first-line treatment^8^.

However, the acquisition of a secondary EGFR mutation, C797S, has been reported in refractory tumors of patients with EGFR T790M-mediated resistance to gefitinib or erlotinib when treated with osimertinib^9,10^. Further, either loss or maintenance of the EGFR T790M mutation has been observed in tumors displaying acquired resistance to osimertinib when patients were previously treated with first or second generation EGFR-TKIs^11,12^. On the other hand, off-target genetic mutational alterations involving *KRAS, BRAF, PIK3CA, PTEN, CTNNB1, TSC2, RET,* and *FGFR3* are associated with acquired resistance to osimertinib^11–16^. Other off-target mechanisms for osimertinib resistance include gene amplification and/or enhanced expression of *HER2, MET, FGFR, MAPK1, AKT3* and *AXL*, and activation of SRC family kinase (*SFK)/*focal adhesion kinase *(FAK)* and Sonic Hedgehog (*SHH*)^14,16–19^. Among these pleiotropic mechanisms for drug resistance to EGFR-TKIs, activation of AXL via AXL kinase often confers EGFR-TKI resistance in lung cancer cells in vitro^20^ and in patients^21^, and a combination of osimertinib with a multikinase inhibitor cabozantinib of VEGFR, MET, and AXL overcomes resistance in vitro^22^. However, which mechanism or biomarker may play a greater role in the appearance of osimertinib resistant tumors is not fully understood.

To further develop potent therapeutic strategies to overcome osimertinib resistance, we should elucidate which mechanisms could be closely associated with acquisition of osimertinib resistance, and also which drugs could be useful to overcome osimertinib resistance in progressive lung cancer. In this study, we established osimertinib-resistant (OR) cell lines from lung cancer H1975 cells harboring mutEGFR and T790M after chronic exposure to osimertinib in culture. Acquired resistance to osimertinib is induced by off-target SRC activation in close collaboration with enhanced expression of AXL and Cub domain-containing protein 1 (CDCP1), and activation of AKT. We discuss how a novel bypass pathway involving the AXL/CDCP1/SRC/AKT signaling is activated during acquired resistance to osimertinib, and also how this osimertinib resistance can be overcome.

## Results

### Acquired resistance to osimertinib induces constitutive AKT activation when expression of EGFR and other EGFR family proteins is attenuated

We independently established two OR cell lines, OR1 and OR2, from parental H1975 cells harboring L858R and T790M in *EGFR* by stepwise selection following exposure to osimertinib. OR1 and OR2 exhibited approximately 300-fold higher resistance to osimertinib than the parental H1975 cells, when they exhibited only three to fourfold higher resistance to afatinib but not erlotinib (Table 1). KRAS mutation and a representative osimertinib resistance-related mutation (C797S) in the tyrosine kinase domain of EGFR were not detected in either OR cell line.

**Table 1:** Cytotoxicity of erlotinib, afatinib or osimertinib in H1975 and osimertinib-resistant cell lines.

Cell lines	EGFR mutations	Selected drug	IC_50_ (µmol/L)^a^		
			Erlotinib	Afatinib	Osimertinib
H1975	L858R + T790M		5.48 (1.00)	0.21 (1.00)	0.014 (1.00)
H1975/OR1		Osimertinib	6.30 (1.15)	0.94 (4.48)	4.0 (286)
H1975/OR2		Osimertinib	6.97 (1.27)	0.66 (3.14)	4.04 (289)

^a^The relative resistance, defined as the IC_50_ value divided by the IC_50_ value of the parental cells, is shown in parentheses.

**Figure 1:**
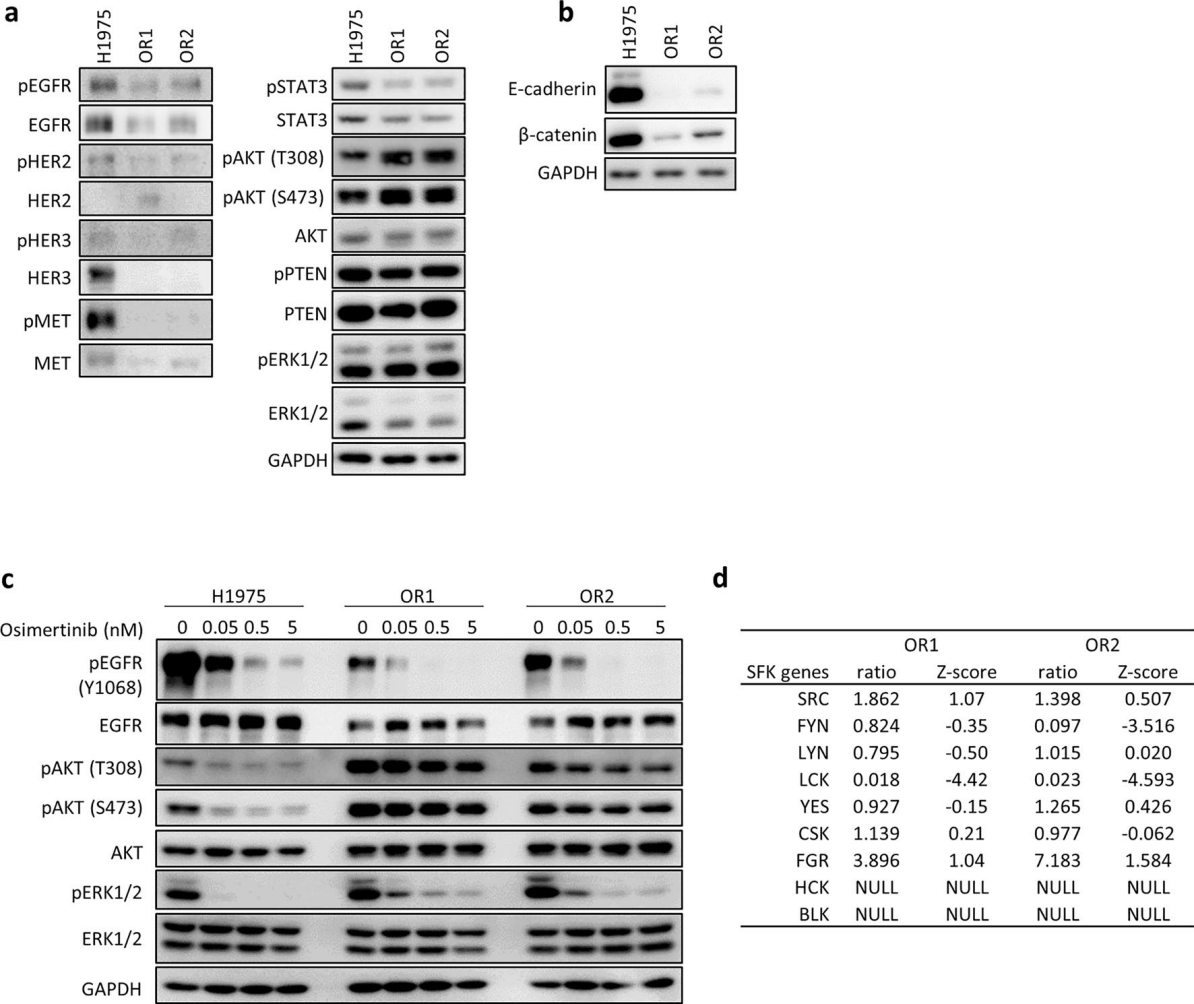
Osimertinib-resistant cell lines, OR1 and OR2, show reduced EGFR expression with constitutive activation of AKT. (**a**) Western blot analysis of EGFR and other molecules in lysates of H1975, OR1 and OR2 cells. (**b**) Western blot analysis of E-cadherin and β-catenin in lysates of H1975, OR1 and OR2 cells. (**c**) Expression levels of EGFR and other molecules analyzed after treatment with various doses of osimertinib for 6 h. (**d**) mRNA expression levels of 9 SFK genes by microarray analysis. Relative-fold changes (OR1 or OR2 vs H1975 cells) are presented with expression levels of each gene in H1975 cells normalized to 1.0. NULL, no significant expression.

OR1 and OR2 displayed markedly reduced expression of EGFR, HER2, HER3, and MET as well as their phosphorylated forms as compared to H1975 cells (Fig. 1a). Among downstream signaling molecules of growth factor receptors, pAKT expression was increased, whereas pSTAT3 expression was reduced, in both OR1 and OR2 cells as compared to H1975 (Fig. 1a). OR1 and OR2 showed markedly decreased expression of E-cadherin and β-catenin as compared to H1975 (Fig. 1b). Osimertinib suppressed phosphorylation of EGFR and ERK1/2 in OR1 and OR2 as H1975 at similar levels. However, phosphorylation of AKT was not affected in OR cells (Fig. 1c).

### Enhanced expression of *AXL, CDCP1,* and *SRC* genes during acquirement of osimertinib resistance

We investigated whether any receptor tyrosine kinase (RTK) and/or membranous glycoproteins were upregulated, and also whether some might be involved in acquired resistance to osimertinib by OR cells. Microarray analysis revealed that 277 relevant genes were up-regulated in both OR1 and OR2. OR1 and OR2 exhibited approximately four to tenfold higher expression of *AXL, PDGFRβ, TGFβR3* and *CDCP1* genes compared to H1975 cells. Cellular sensitivity to various PDGFR kinase inhibitors such as AG1296, SU11652, PDGFR tyrosine kinase inhibitor IV, PDGFR tyrosine kinase inhibitor V, pazopanib, and sorafenib, was similar in H1975 and OR cells (Supplementary Fig. S1, Supplementary Table S1). Furthermore, cellular sensitivity to pan-TGFβR inhibitor (SB431542) and TGFβR1 inhibitor (TGFβR1 kinase inhibitor II) was also similar when assayed using the SCADS inhibitor kit (Supplementary Fig. S1). Enhanced expression of PDGFRβ or TGFβR3 may not be involved in novel activation of cell growth/survival signaling pathways by osimertinib resistance. On the other hand, previous studies have reported that enhanced AXL expression is often associated with acquired resistance to osimertinib in lung cancer cells^21,22^, and also that enhanced expression of both AXL and CDCP1 is associated with poor outcomes of patients with NSCLC^23^. We further focused on AXL and CDCP1 to examine whether enhanced expression of these molecules is specifically associated with osimertinib resistance.

EGFR-TKI-resistant lung cancer cells demonstrated that activation of off-target signaling molecules such as SRC, FAK, and YES, conferred resistance to various EGFR-TKIs^17,24,25^. Additionally, osimertinib resistance can be overcome using combinations of drugs targeting SFK and FAK^17,24,25^. Seven SFK-related genes have been identified as genomic modifiers of EGFR dependence in lung cancer cells harboring mutEGFR^26^. Secondly, we examined whether expression of SFK genes is involved in osimertinib resistance. Microarray analysis revealed enhanced expression of *FGR* and *SRC* mRNAs in OR1 and OR2 cells as compared to H1975 (Fig. 1d).

### AXL activation supports acquired resistance to osimertinib in collaboration with SRC

**Figure 2:**
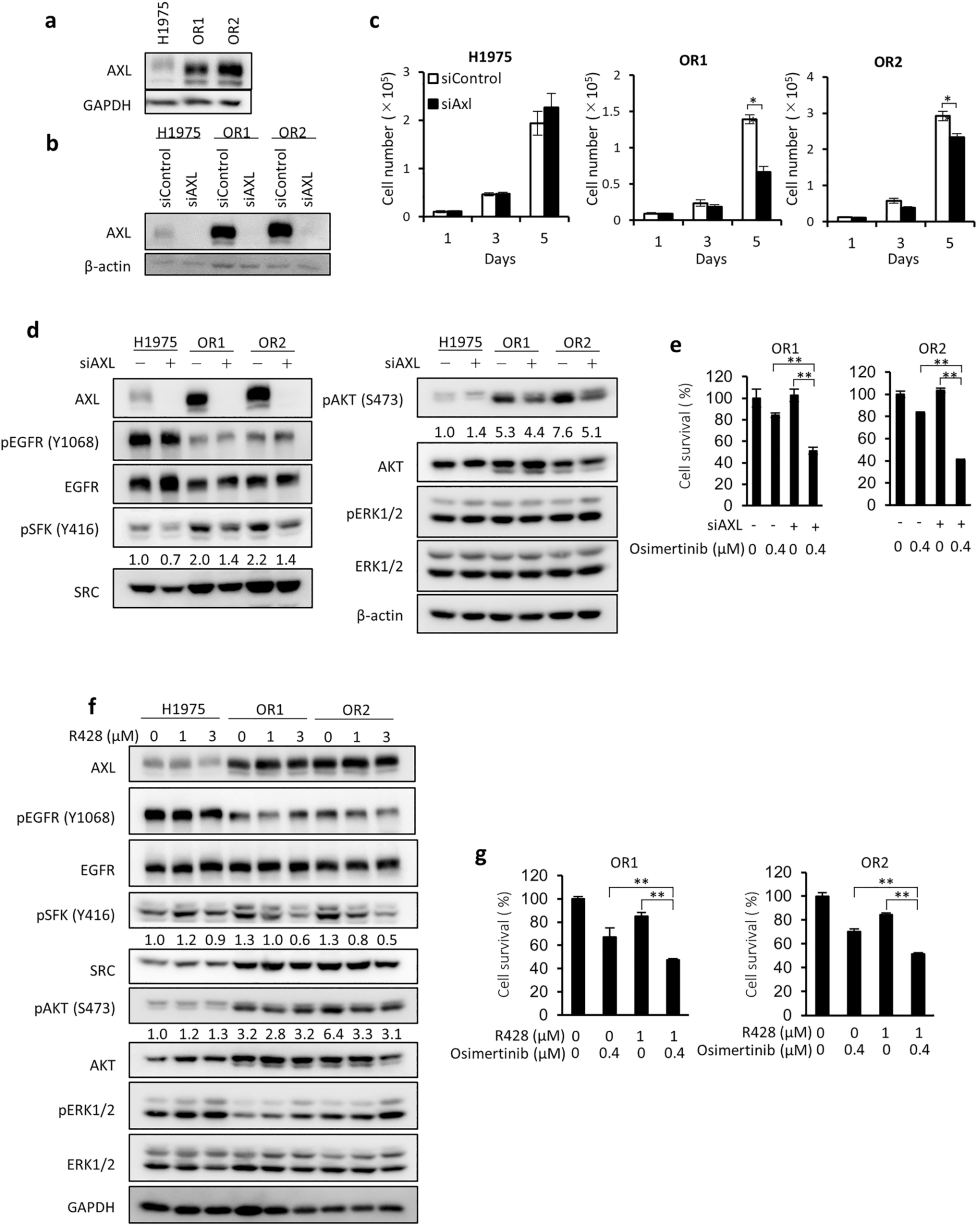
Enhanced expression of AXL is closely associated with OR1 and OR2 cell growth. (**a**) Protein expression levels of AXL in H1975, OR1 and OR2 cells by western blot analysis. (**b**) AXL protein expression levels in three cell lines treated with control or *AXL* siRNA for 3 days. (**c**) Effects of AXL silencing on growth of three cell lines. Each value is the mean ± SD of triplicate dishes. *P < 0.05, **P < 0.01, two-tailed Student’s *t*-test. (**d**) Effects of AXL silencing on expression of pSFK, pAKT, and pERK in the three cell lines. Cell were treated with an *AXL* siRNA for 3 days followed by western blot analysis. (**e**) Combination of osimertinib and *AXL* siRNA on OR1 and OR2 cell growth. Cells were exposed to 0.4 μM osimertinib and/or *AXL* siRNA for 3 days. Each value is the mean ± SD of triplicate dishes. *P < 0.05, **P < 0.01. (**f**) Expression levels of pSFK, pAKT, and pERK were analyzed after treatment with various doses of R428 for 2 days. (**g**) Sensitivity to osimertinib with or without R428. Cells were exposed for 3 days and subjected to a water-soluble tetrazolium salt (WST) assay. Each value is the mean ± SD of triplicate dishes.

Enhanced expression of *AXL* induces resistance to EGFR-TKIs and activates PI3K/AKT and MAPK/ERK pathways^20,22^. As shown in Fig. 2a, expression of AXL protein was also higher in OR1 and OR2 than H1975 cells. siRNA mediated AXL silencing suppressed growth of OR1 and OR2 cells by approximately 20% to 50%, but did not suppress growth of H1975 cells at all (Fig. 2b,c), and suppressed phosphorylation of AKT and SFK (Fig. 2d). Combination therapy with *AXL* siRNA augmented the cytotoxicity of osimertinib in both OR1 and OR2 cells (Fig. 2e). Furthermore, R428, a kinase inhibitor of AXL, suppressed phosphorylation of SFK in OR1 and OR2 cells, but not in H1975, where the inhibitory effect of R428 on AKT phosphorylation was only slight in OR1, OR2, and H1975 cells (Fig. 2f). Combination with R428 also augmented the cytotoxicity of osimertinib in OR1 and OR2 cells (Fig. 2g). AXL is thus likely to be involved, at least in part, in acquired resistance to osimertinib by its association with SFK activation.

### CDCP1 activation also supports acquired resistance to osimertinib in collaboration with SRC activation

**Figure 3:**
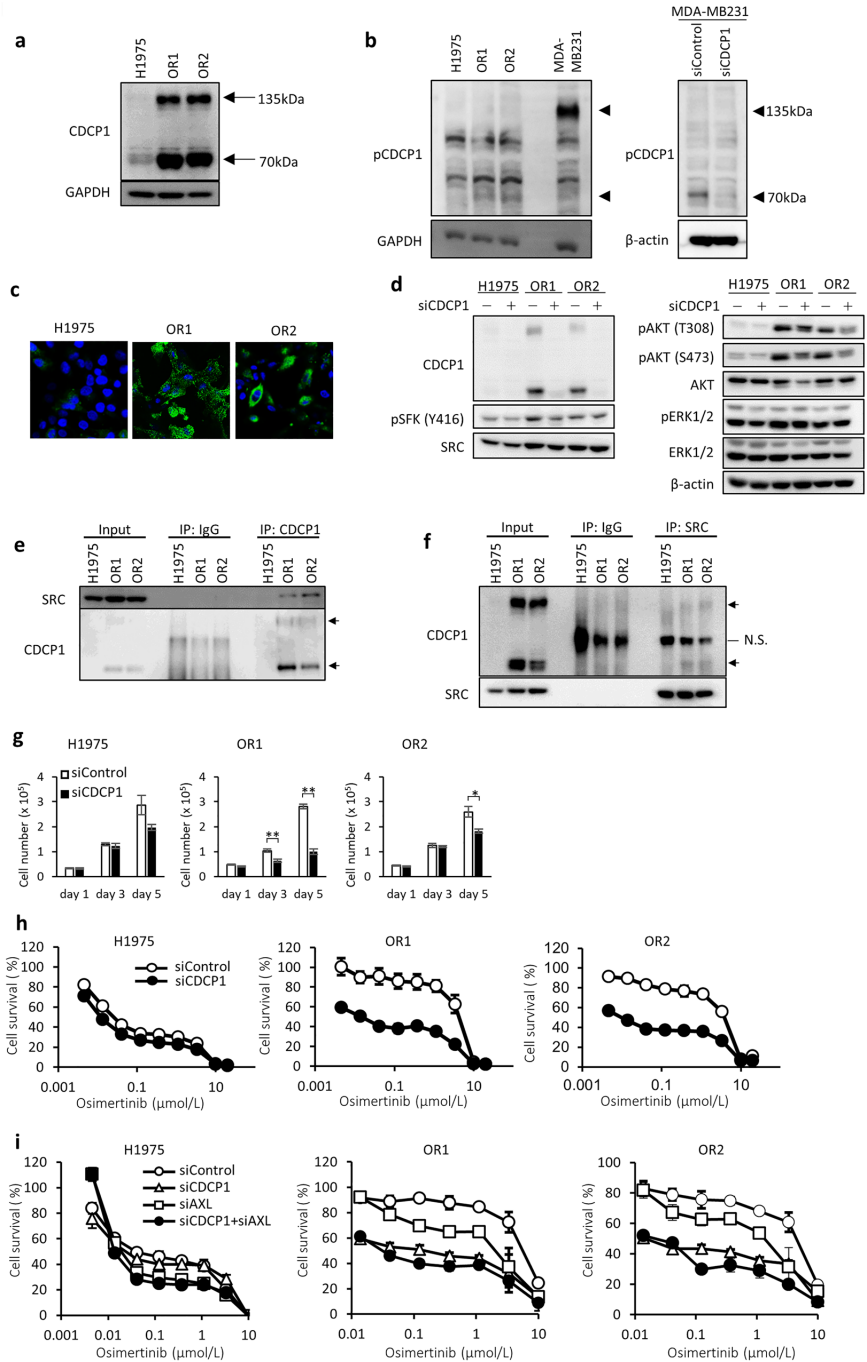
Enhanced expression of CDCP1 in OR1 and OR2 cells is closely associated with cell growth and activation of SFK and AKT. (**a**) Expression levels of CDCP1 protein in three cell lines by western blot analysis. Arrows indicate two forms (135 kDa and 70 kDa) of CDCP1 protein. (**b**) Expression levels of phosphorylated CDCP1 protein in three cell lines and effects of CDCP1 silencing on phosphorylation of CDCP1 by western blot analysis. Arrows indicate two forms (135 kDa and 70 kDa) of phosphorylated CDCP1 protein. MDA-MB231 cells were used as positive control. (**c**) Immunofluorescence analysis of CDCP1 in H1975, OR1, and OR2 cells. (**d**) Effects of CDCP1 silencing on activation of SFK (pSFK) and AKT (pAKT) by western blot analysis when treated with siRNA for 4 days. (**e,f**) Co-immunoprecipitation assays to determine interactions of SRC and CDCP1. SRC was detected after immunoprecipitation with anti-CDCP1 antibody (**e**), and CDCP1 was detected after immunoprecipitation with anti-SRC antibody (**f**). (**g**) Effects of CDCP1 silencing on growth of three cell lines. Each value is the mean ± SD of triplicate dishes. *P < 0.05, **P < 0.01, two-tailed Student’s *t*-test. (**h**) Effects of combination of osimertinib and CDCP1 silencing on cell growth in three cell lines. Cells were exposed to various doses of osimertinib with or without *CDCP1* siRNA for 3 days, followed by WST assays. Each value is the mean ± SD of triplicate dishes. (**i**) Combination effect of both siRNAs of CDCP1 and AXL on cell growth in the presence of osimertinib. Cells were exposed to various doses of osimertinib for 3 days, followed by WST assays. Each value is the mean ± SD of triplicate dishes.

CDCP1 promotes cell growth and survival signaling pathways through EGFR, HER2, integrinβ1, SFK, and PKCδ^23,27–29^. Full length CDCP1 (135 kDa) and its cleaved form (70 kDa) are phosphorylated by SFKs followed by interaction with other RTKs and/or non-RTKs^23,27–31^. Uekita et al. previously reported that H1975 cells harboring wild type KRAS showed relatively lower expression of CDCP1 when mRNA expression levels of CDCP1 were analyzed in a panel of 58 NSCLC cell lines^32^. Immunoblot analysis demonstrated markedly higher expression of both full-length and cleaved CDCP1 in OR1 and OR2 cells than H1975 cells (Fig. 3a). The cleaved form of CDCP1 was apparently phosphorylated in both OR1 and OR2 cells (Fig. 3b). Immunocytochemical analysis also revealed markedly enhanced expression of CDCP1 in cytoplasm and membrane fractions of OR1 and OR2 cells (Fig. 3c).

We examined whether enhanced CDCP1 expression was associated with activation of SRC and AKT in OR cells (Fig. 3d). CDCP1 silencing reduced expression of pSFK and pAKT by OR1 and OR2 cells (Fig. 3d). Immunoblot assays showed protein–protein interactions involving CDCP1 and SRC in OR cells after immunoprecipitation with anti-CDCP1 antibody (Fig. 3e), or when immunoprecipitated with anti-SRC antibody (Fig. 3f). CDCP1 silencing significantly induced growth suppression of OR1 and OR2 cells by approximately 30% to 70% of the control, and that of H1975 cells by approximately 30% (Fig. 3g). CDCP1 silencing also enhanced susceptibility to osimertinib in OR1 and OR2 cell (Fig. 3h). We further examined whether silencing of both CDCP1 and AXL enhance the sensitivity to osimertinib additively or synergistically as compared with silencing of each CDCP1 or AXL alone (Fig. 3i). Silencing of both CDCP1 and AXL increased osimertinib sensitivity additively rather than synergistically in OR1 and OR2 cells (Fig. 3i).

### Enhanced expression of SRC supports acquired resistance to osimertinib and collateral sensitivity to the SRC inhibitor dasatinib

**Figure 4:**
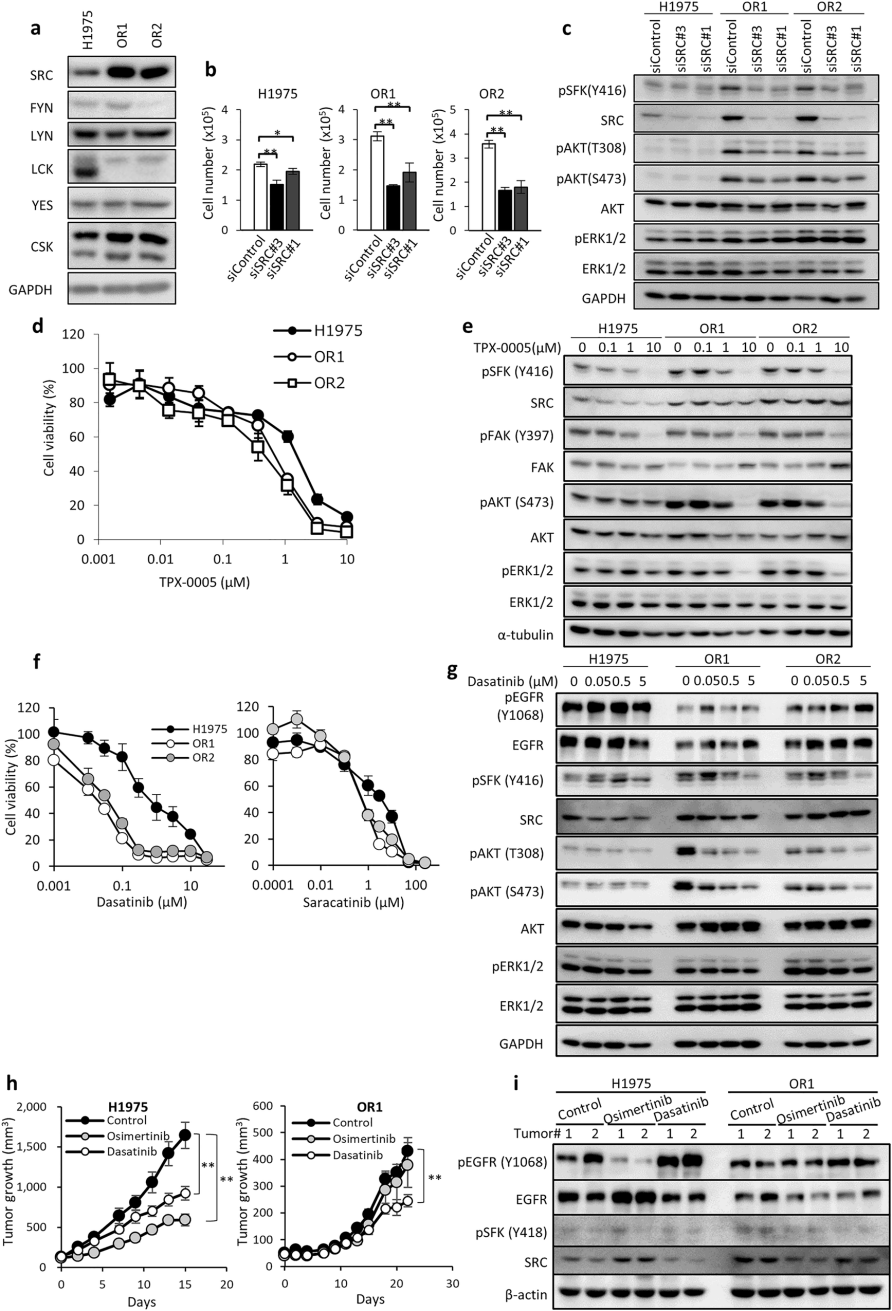
Osimertinib-resistant cells exhibit enhanced SRC expression, growth dependency on SRC, and collateral sensitivity to dasatinib. (**a**) Western blot analysis showing 6 *SFK* genes in three cell lines. (**b**) SRC knockdown induced cell growth inhibition at differential levels in H1975, OR1, and OR2 cells when treated with siRNAs for 4 days. Each bar shows the mean ± SD of triplicate wells, *P < 0.05, **P < 0.01, two-tailed Student’s *t*-test. (**c**) Effect of *SRC* siRNA on activation of SFK and AKT analyzed after treatment with *SRC* siRNAs for 3 days. GAPDH served as loading control. (**d**) Dose response curves of three cell lines to TPX-0005 when treated for 3 days. Each value is the mean ± SD of triplicate dishes. (**e**) Western blot analysis showing pSFK, pFAK, pAKT, and pERK in H1975, OR1 and OR2 cells when treated with TPX-0005 for 6 h. (**f**) Dose response curves of H1975, OR1, and OR2 cells to various doses of dasatinib and saracatinib when exposed for 3 days. Each value is the mean ± SD of triplicate dishes. (**g**) Western blot analysis showing EGFR and other molecules when treated with various doses of dasatinib for 6 h. (**h**) Animals bearing H1975 or OR1 xenografts were treated with osimertinib and dasatinib. Results are shown as mean tumor volumes and SD at each timepoint. **P < 0.01, two-tailed Student’s *t*-test. (**i**) Western blot analysis of H1975 and OR1 tumors after 15 (H1975) and 22 (OR1) days of treatment with osimertinib or dasatinib.

Expression of *SFK* mRNA was augmented in OR1 and OR2 cells as compared to H1975 (see Fig. 1d). Among SFK proteins (SRC, FYN, LYN, LCK, and YES), expression level of SRC was higher in OR1 and OR2 than H1975 cells (Fig. 4a), consistent with microarray analysis (Fig. 1d). However, expression of FGR was not detected when determined by immunoblot analysis (data not shown). To further examine the effects of SRC on proliferation of OR cells, we performed siRNA-mediated knockdown of the *SRC* gene. Silencing of SRC suppressed cell growth significantly by approximately 50% accompanied by suppression of AKT and SFK phosphorylation in OR1 and OR2 cells (Fig. 4b,c). On the other hand, cell growth suppression of H1975 was by 10% to 20% (Fig. 4b). TPX-0005 is an inhibitor of SRC/FAK, and a phase 1/2 clinical trial of TPX-0005 is on-going for patients with advanced solid tumors^23,33^. OR1 and OR2 cells displayed three to fivefold higher sensitivity to TPX-0005 than H1975 cells (Fig. 4d). Phosphorylation of SFK and FAK was suppressed at similar levels in all three cell lines by TPX-0005 up to 1 µM, and TPX-0005 at 10 µM inhibited phosphorylation of both AKT and ERK in OR cells, but not in H1975 cells (Fig. 4e).

OR1 and OR2 showed collateral sensitivity to TPX-0005 (Fig. 4d), and we further examined their cellular sensitivity to inhibitors of SRC and other targets. OR cells exhibited similar sensitivity to inhibitors of MET/ALK, IGF-1R, and PDGFR as H1975 cells (Supplementary Table S1). However, surprisingly, both OR1 and OR2 cells showed 10- to 50-fold higher collateral sensitivity to dasatinib and also three to sixfold higher sensitivity to other SFK inhibitors, including saracatinib and PP1, than H1975 cells (Fig. 4f, Supplementary Table S1). SRC may thus be activated by osimertinib resistance, conferring enhanced susceptibility to SFK inhibitors. We next examined whether dasatinib differentially inhibited AKT phosphorylation in H1975 and OR cells. Dasatinib at 0.5 to 5 μmol/L inhibited phosphorylation of AKT and SFK in OR1 and OR2 cells, but only slightly (if at all) in H1975 cells (Fig. 4g). Both OR1 and OR2 showed approximately threefold higher sensitivity to mTOR inhibitor AZD8055 than H1975 (Supplementary Table S1). However, there was no apparent difference in the effect of AZD8055 on the phosphorylation levels of both mTORC1/C2 downstream molecules, AKT and S6K, among H1975, OR1 and OR2 cells (Supplementary Fig. S2), suggesting that underlying mechanism why different sensitivity to mTOR inhibitor remains to be further studied.

We further examined the antitumor effects of dasatinib on OR cells in vivo. Treatment with dasatinib resulted in antitumor effects in OR1 and H1975 cells. It was noted that the inhibitory effect of dasatinib on OR1 tumor growth was much greater than that of osimertinib, whereas the antitumor effects of dasatinib and osimertinib on H1975 tumor growth was comparable (Fig. 4h). Western blotting revealed osimertinib suppressed EGFR phosphorylation only in H1975 tumors, but not in OR tumors (Fig. 4i), and dasatinib suppressed SRC phosphorylation in OR tumors compared to H1975 tumors (Fig. 4i). Together, OR cells thus presented collateral sensitivity to dasatinib in vivo as well as in vitro.

### AXL and CDCP1 expression is induced by short term exposure to osimertinib in H1975 cells

**Figure 5:**
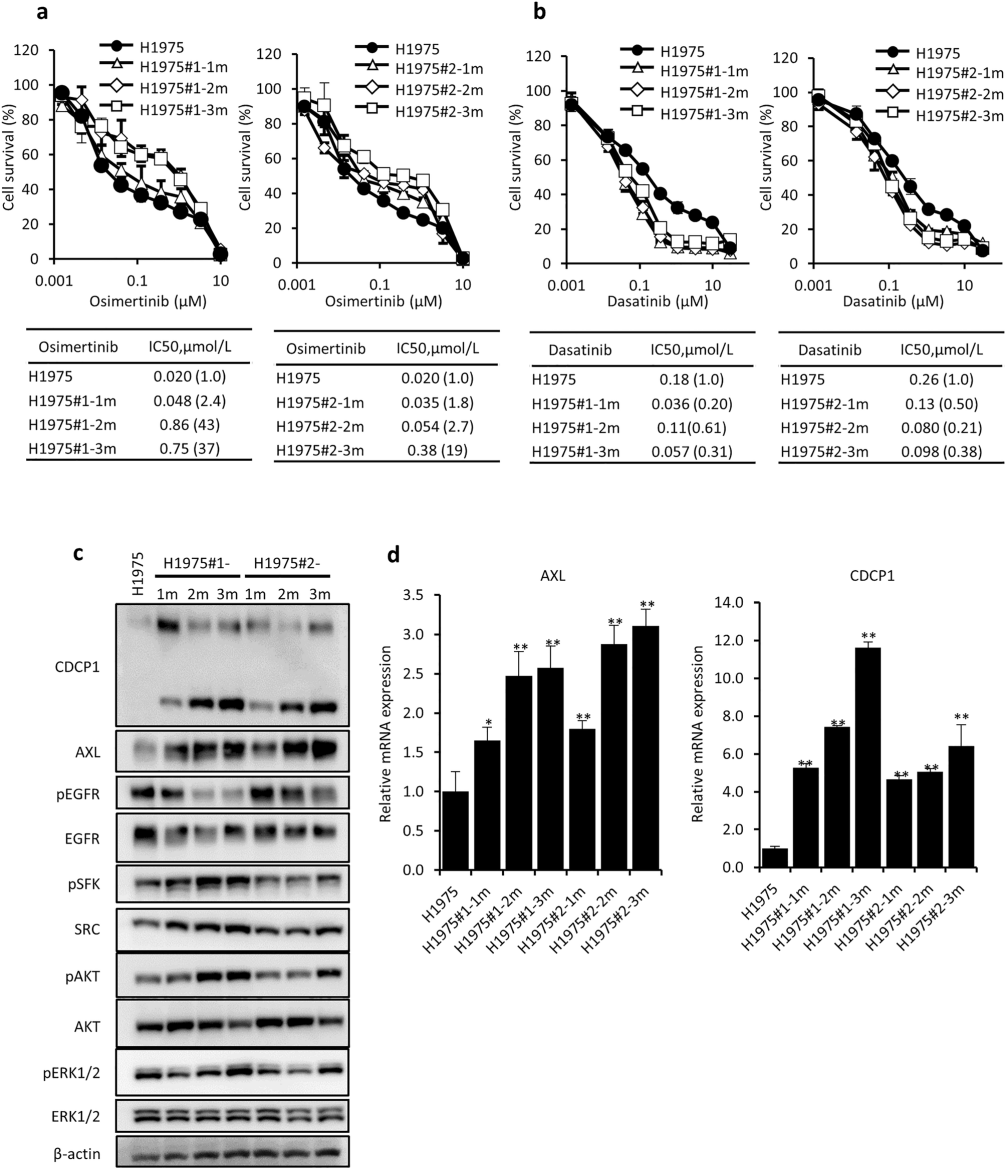
Acquisition of osimertinib resistance and expression of AXL and CDCP1 by H1975 cells exposed for 1, 2, and 3 months to osimertinib. (**a,b**) Cellular sensitivity to osimertinib (**a**) and dasatinib (**b**) of H1975#1, H1975#1-1m, H1975#1-2m and H1975#1-3m, and H1975#2, H1975#2-1m, H1975#2-2m and H1975#2-3m cells. (**c**) Expression of AXL, CDCP1, EGFR, pEGFR, pSFK, and pAKT detected by western blot analysis. (**d**) Expression of *AXL* and *CDCP1* mRNA by RT-qPCR analysis. Each value is the mean ± SD of triplicate dishes. *P < 0.05, **P < 0.01 vs H1975 (one-way ANOVA, Dunnett’s test).

We next examined when drug resistance as well as enhanced expression of AXL and CDCP1 are induced during short term exposure to osimertinib. OR1 and OR2 cells were independently established after exposure to the drug for 4 months, from two different flasks H1975#1 and H1975#2, respectively. We first examined cellular sensitivity to osimertinib and dasatinib using frozen stocks of H1975#1 and H1975#2 cells when exposed to osimertinib for 1 month (H1975#1-1m and H1975#2-1m), 2 months (H1975#1-2m and H1975#2-2 m), and 3 months (H1975#1-3m and H1975#2-3m). More than tenfold greater resistance to osimertinib was acquired after exposure for 2 or 3 months (Fig. 5a), whereas H1975#1-1 m and H1975#2-1m cells showed collateral sensitivity to dasatinib (Fig. 5b). Expression of CDCP1 and AXL was enhanced in H1975#1 and H1975#2 cells when exposed for 1 month, accompanied by enhanced activation of SFK and AKT (Fig. 5c). Expression of both *AXL* and *CDCP1* mRNAs was also augmented in H1975#1 and H1975#2 cells when exposed for 1 month (Fig. 5d). Enhanced expression of both AXL and CDCP1 is thus likely to be closely associated with acquisition of drug resistance.

### Enhanced AXL and CDCP1 expression in refractory tumors of patients treated with EGFR-TKIs

**Figure 6:**
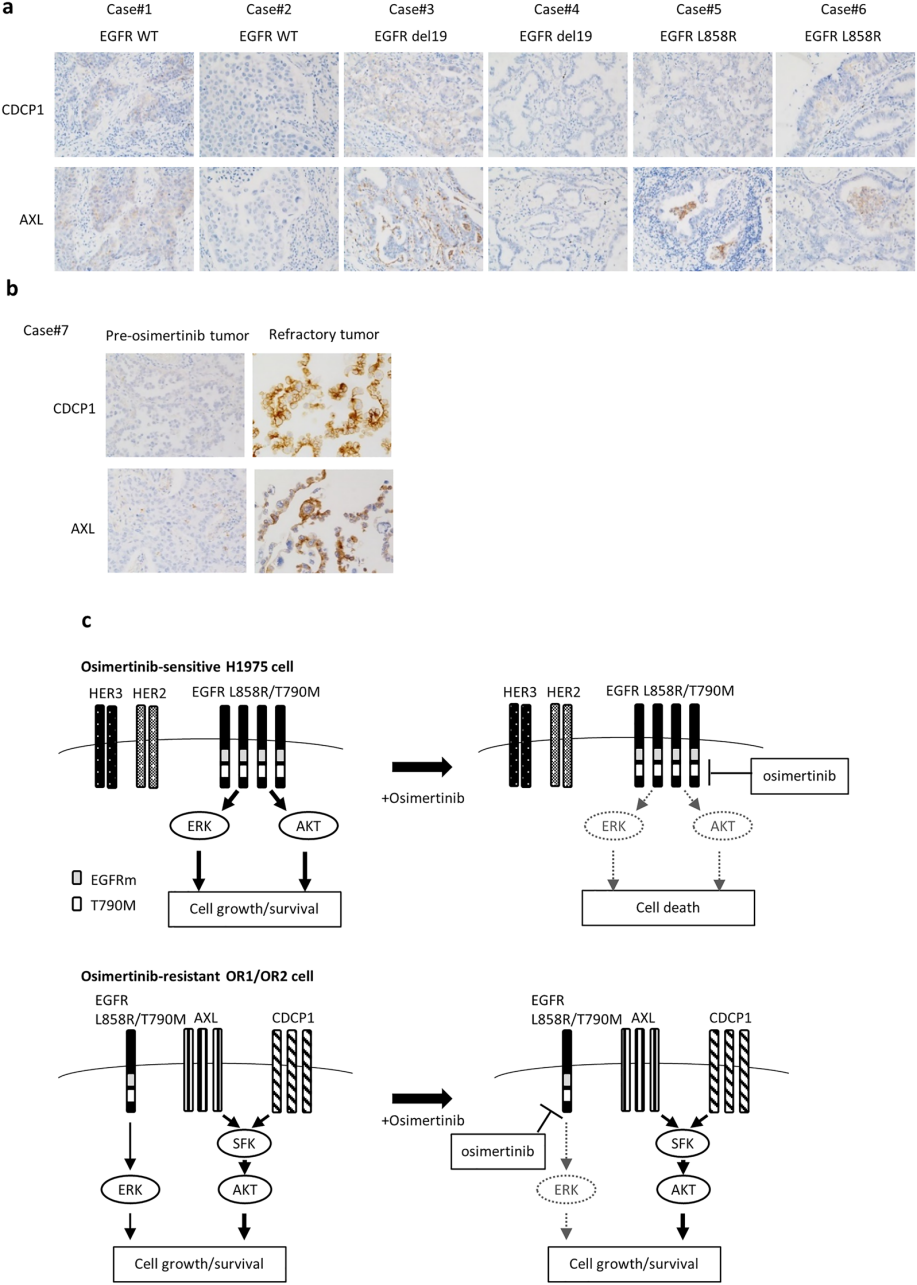
Expression of AXL and CDCP1 in lung tumors of patients. (**a**) IHC analysis of AXL or CDCP1 expression in surgically resected tumors of 6 patients (cases #1–6) before chemotherapy. (**b**) IHC analysis of AXL and CDCP1 in tumors before EGFR TKI treatment and after recurrence (cases #7). (**c**) Hypothetical model of how osimertinib resistance is acquired. In osimertinib-sensitive cells, the PI3K/AKT pathway driven by EGFR harboring mutEGFR and T790M is highly susceptible to osimertinib. By contrast, in osimertinib resistant cells, expression of the driver EGFR is reduced, accompanied by SRC activation together with enhanced expression of AXL and CDCP1. Enhanced expression of AXL and CDCP1 activates SFK and AKT signaling pathway, resulting in promotion of cell growth and survival.

Expression of AXL and CDCP1 in tumor samples was examined by immunohistochemical (IHC) analysis after tumors were surgically resected in 6 patients (#1, #2 with wild-type EGFR; #3, #4 with *EGFR*del19; #5, #6 with L858R EGFR) before chemotherapy (Fig. 6a). All six tumors showed no (score 0) or very low (score 1) expression of AXL and CDCP1. We further assessed whether expression of AXL and/or CDCP1 was altered in refractory tumors of patients. One clinical tumor sample involving pre-and post-therapeutic treatment with osimertinib (#7) were analyzed (Fig. 6b). There was only slight (if any) expression of AXL and CDCP1 (score 0 or 1) in tumors of a patient before treatment. However, there was markedly elevated expression of both AXL and CDCP1 in recurrent tumor after treatment with osimertinib (Fig. 6b). Further analysis with increasing number of recurrent tumors should be required to evaluate whether enhanced co-expression of both AXL and CDCP1 is useful to predict recurrence after treatment with osimertinib.

## Discussion

We characterized osimertinib resistance by enhanced co-expression of AXL and CDCP1 together with activation of SRC and AKT. Furthermore, we presented our hypothetical model of how enhanced expression of AXL and CDCP1 plays a key role in acquired resistance to osimertinib in collaboration with activation of SRC and AKT (Fig. 6c). Expression of both *AXL* and *CDCP1* was markedly augmented in OR1 and OR2 cells and silencing of each AXL or CDCP1 gene potentiate cell growth inhibition by osimertinib in OR1 and OR2. Karachaliou et al. reported that co-expression of AXL and CDCP1 is closely associated with poor outcomes in patients with NSCLC^23^. Co-targeting EGFR, STAT3, and SRC-YAP1 is highly synergistic in vitro and in vivo^23,34,35^. EGFR-independent cell growth/survival is switched to being SRC-dependent in EGFR-TKI-resistant cells, and SFK inhibitors including dasatinib overcome resistance to EGFR-TKI when combined with gefitinib^36^, afatinib^24^, and also osimertinib^37^. We demonstrated that OR1 and OR2 cells exhibit activation of SRC and AKT signaling pathways, and hypersensitivity to dasatinib and other SFK inhibitors, consistent with the notion that combinations of SFK inhibitors represent a useful therapeutic approach to overcome osimertinib resistance^17^.

AXL is overexpressed or reactivated by acquired resistance to osimertinib^21,22^, and AXL is associated with the appearance of drug resistant tumors and poor survival in patients with NSCLC^20^. Targeting AXL overcomes acquired resistance to osimertinib and other EGFR-TKIs^38,39^, and multiple AXL inhibitors have been developed and advanced to clinical trials involving cancer therapy to overcome drug resistance^40,41^. GAS6/AXL signaling potently activates SRC and MET in cancer cells^42^, and treatment with SFK inhibitors, dasatinib and PP2, blocks GAS6/AXL signaling-dependent expression of S100A10 by cancer cells^43^. We demonstrated that AXL knockdown or R428 treatment induced greater specific inhibition of cell growth and phosphorylation of AKT and SFK in OR cells than H1975 cells (Fig. 2). Together, AXL-induced growth signaling was associated with SRC and AKT activation in osimertinib-resistant lung cancer (Fig. 6c).

In this present study, expression of CDCP1 was also markedly augmented together with that of AXL in osimertinib resistant cell lines and also in refractory tumors appearing after treatment with osimertinib (Figs. 2a, 3a, 6b). Growth of OR1 and OR2 cells was highly susceptible to the inhibitory effects of CDCP1 knockdown as compared to H1975 cells when combined with osimertinib (Fig. 3g,h,i). This is the first study to reveal the association of CDCP1 with acquired resistance to osimertinib in cancer cells. Recent prospective proteomics analysis by Dagnino et al. has demonstrated that higher blood levels of CDCP1 protein are clearly associated with the risk of lung cancer^44^, suggesting the potential role of CDCP1 in onset of lung cancer. On the other hand, CDCP1 has been reported to enhance WNT/β-catenin signaling pathway through increased nuclear localization of β-catenin^45^. OR1 and OR2 cells showed decreased expression of E-cadherin, which is a WNT/β-catenin signaling regulated gene (Fig. 1b). However, β-catenin expression was also decreased (Fig. 1b), suggesting that CDCP1 was less associated with activation of WNT/β-catenin signaling pathway in OR1 and OR2 cells.

Relevant studies previously demonstrated that cleavage of CDCP1 induces formation of CDCP1/SRC/PKCδ complexes^31^. CDCP1 is phosphorylated by SFK and CDCP1 phosphorylation mediates PKCδ recruitment^46^. Overexpression of CDCP1 results in induction of SFK and AKT phosphorylation^31,47^. Consistent with these studies, CDCP1 was phosphorylated (Fig. 3b), and CDCP1 protein interacted with SRC protein in OR1 and OR2 cells (Fig. 3e,f). Furthermore, CDCP1 knockdown also induced greater inhibition of phosphorylation of SFK and AKT in OR1 and OR2 cells than H1975 cells (Fig. 3d). Cleavage of CDCP1 and phosphorylation of cleaved CDCP1 may be required for AKT phosphorylation in osimertinib resistant cells after formation of CDCP1 and SRC complexes. Further study should be required to understand how CDCP1 activates SFK and AKT at molecular basis. A previous study by Uekita et al. demonstrated that oncogenic RAS/ERK signaling activated CDCP1 to promote tumor invasion and metastasis in lung cancer^32^. Together with our present study, CDCP1 may be required for the functional link between RAS and SRC signaling during development of malignancies including drug resistance to osimertinib.

A clinical study has reported that HER3 expression was augmented in patients with lung tumors refractory to treatment with EGFR-TKIs. Furthermore, HER3 was augmented through inhibition of PI3K/AKT/mTOR signaling after treatment with osimertinib for 24 h in NSCLC cell lines including H1975^48^. In our present study, OR1 and OR2 cells, which were established after long exposure to osimertinib, showed decreased expression of HER3 (Fig. 1a). By contrast, phosphorylation of AKT was increased in both OR1 and OR2 cells as compared to H1975 (Fig. 1a). Activated AKT via AXL/CDCP1/SRC axis may reduce cell growth dependency on HER3 in OR1 and OR2 cells. However, since this is highly speculative, further study should be required to explore the correlation among AXL, CDCP1, AKT and HER3 in osimertinib resistant lung tumor.

In conclusion, our present study revealed a novel mechanism in that acquired resistance to osimertinib conferred cell growth dependence on AXL and CDCP1 in collaboration with SRC-induced bypass activation pathway when EGFR-dependent on-target signals are attenuated. Additionally, SRC activation further induced the AKT signaling pathway, accompanied by concomitant collateral sensitivity to SRC targeted drugs. Osimertinib resistance is thus independently mediated through both AXL and CDCP1, together with activation of SRC-induced bypass signals, indicating that combination therapy involving receptor tyrosine kinase inhibitors and non-receptor tyrosine kinase inhibitors targeting AXL, CDCP1, and SRC can overcome osimertinib resistance.

## Methods

### Cell culture and establishment of OR cells from H1975 cells

H1975 cells were purchased from American Type Culture Collection We independently established two OR clones (OR1 and OR2) by step-wise increasing doses of osimertinib up to 2 μmol/L over a period of four months. These cells were maintained in RPMI medium supplemented with 10% fetal bovine serum (FBS) and incubated in a humidified atmosphere of 5% CO_2_ at 37℃. All cell lines were passaged for ≤ 6 months and were not further tested or authenticated by the authors. Osimertinib resistance was stably maintained during cell culture for six months in the absence of drug. EGFR C797S and KRAS mutation was analyzed using Sanger sequencing (Fasmac DNA Sequence Service, Fasmac Co. Ltd.). To analyze the mutations, exon 20 of the EGFR gene was amplified using the forward primer (5′-CATTCATGCGTCTTCACCTG-3′) and the reverse primer (5′-CATATCCCCATGGCAAACTC-3′), and exon 1 of the KRAS gene was amplified using the forward primer (5′-CGTCGATGGAGGAGTTTGTA-3′) and the revere primer (5′-GGACCCTGACATACTCCCAA-3′).

### Reagents

Erlotinib was kindly provided by F. Hoffman-La Roche Ltd; TPX-0005 was from TP Therapeutics, Inc.; dasatinib was purchased from Bio Vision; sorafenib was from Toronto Research Chemicals Inc; the other inhibitors were from Selleck Chemicals. Anti-HER2 and anti-pHER2 antibodies were purchased from Upstate Biotechnology; anti-EGFR, anti-pEGFR, anti-pHER3, anti-MET anti-pMET, anti-ERK1/2, anti-pERK1/2, anti-AKT, anti-pAKT (T308 or S473), anti-STAT3, anti-pSTAT3, anti-PTEN, anti-AXL, anti-CDCP1, anti-pCDCP1, anti-SRC, anti-FYN, anti-LYN, anti-YES, anti-LCK, anti-pSFK (Y416), anti-β-catenin, anti-S6K, and anti-pS6K antibodies were from Cell Signaling Technology; anti-E-cadherin antibody was from BD biosciences; anti-HER3 antibody was from Santa Cruz Biotechnology; anti-β-actin antibody was from Abcam, Inc.; Anti-GAPDH antibody was from PROMEGA; α-tubulin antibody was sourced from Sigma-Aldrich.

### Western blot analysis

The cells were rinsed in ice-cold phosphate-buffered saline and lysed in RIPA buffer containing 1 mM phenylmethylsulfonyl fluoride, 10 µg/mL aprotinin, 10 µg/mL leupeptin, and 1 mM sodium orthovanadate. The cell lysates were separated through sodium dodecyl sulfate–polyacrylamide gel electrophoresis and transferred to Immobilon membranes (Millipore Corp). After transfer, membranes were incubated in a 5% skim milk. They were subsequently probed with antibodies, washed, and visualized by using horseradish peroxidase-conjugated secondary antibodies (Cytiva). The original images are presented in Supplementary information file. Full-length images could not be shown, because the membranes were cut to appropriate size prior to hybridization with various antibodies.

### Co-immunoprecipitation assay

Cells were rinsed in ice-cold PBS and lysed in IP lysis buffer (50 mM Tris–HCl (pH 8.0), 250 mM NaCl, 1 mM EDTA, 10% glycerol, 0.3% NP-40). The extract was incubated overnight at 4 ℃ with antibody against SRC or antibody targeting CDCP1 (Cell Signaling Technology) in NET-gel buffer (50 mM Tris–HCl (pH 7.5), 150 mM NaCl, 1 mM EDTA, 0.25% gelatin, 0.02% sodium azide, 1 mM PMSF, 10 mg/mL leupeptin, and 10 mg/mL aprotinin), and then with Protein A/G PLUS-Agarose (Santa Cruz Biotechnology) for 1 h at 4 ℃. The beads were washed twice with NET-gel buffer and once with 10 mM Tris–HCl (pH 7.5) 0.1% NP40. After centrifugation, the precipitate and starting material were boiled in SDS-PAGE sample buffer and western blot analysis was performed.

### Gene expression microarrays

Complementary RNA was amplified, labeled, and hybridized to GE SuperPrint G3 Human 8 × 60 K microarrays according to the manufacturer’s instructions (Agilent Technologies). All hybridized microarray slides were scanned using an Agilent scanner. Relative hybridization intensities and background hybridization values were calculated using Agilent Feature Extraction Software. To identify up- or down-regulated genes, we calculated ratios (non-log scaled fold-changes) from the normalized signal intensities of each probe for comparison between control and experimental samples. We then established criteria for regulated genes: up-regulated genes, ratio ≥ twofold; down-regulated genes, ratio ≤ 0.5.

### Cytotoxicity assays

Cells were plated in 96-well flat-bottomed plates and cultured for 24 h before exposure to various concentrations of drugs or agents from the Screening Committee of Anticancer Drugs (SCADS) inhibitor kit for 72 h at 37 ℃. 15 μL of Cell Count Reagent SF (Nacalai Tesque) was added to each well and the plates were incubated for a further 2 to 4 h at 37 ℃. Optical absorbance was measured at 450 nm using a 96-well plate reader. Triplicate wells were tested at each drug concentration. IC_50_ values, defined as the drug concentration resulting in a 50% reduction in absorbance, were calculated from survival curves.

### Small interfering RNA transfection

Cells were treated with small interfering RNA (siRNA) duplexes using Lipofectamine RNAiMAX and Opti-MEM medium (Invitrogen) according to the manufacturer’s recommendations. *AXL* siRNA, *SRC* siRNA#3, *SRC* siRNA#1, and *CDCP1* siRNA were purchased from Invitrogen. Non-specific (control) siRNA was purchased from Qiagen.

### Cell growth

For cell growth under AXL, SRC, and CDCP1 knockdown condition, cells were plated in 35 mm dishes and the following day, cells were treated with cognate siRNAs. Cell numbers in each dish were counted using a Z2 Coulter Particle Count and Size Analyzer (Beckman Coulter Inc., CA). Triplicate dishes were tested on each day. Results are expressed as means ± SD of triplicate dishes.

### Animals

Animal experiments were performed with the approval of the Ethics of Animal Experiments Committee at Kyushu University Graduate School of Medical Sciences and according to the ARRIVE guidelines. Male athymic BALB/c nu/nu mice (5 weeks old) were purchased from CLEA and housed in microisolator cages and maintained under a 12-h light/dark cycle. Water and food were supplied ad libitum. All methods were performed in accordance with the relevant guidelines and regulations.

### Mice xenograft

Approximately 5.0 × 10^6^ H1975 or OR1 cells in 200 µL of 50% Matrigel were implanted into the subcutaneous tissue of the right abdominal wall of the mice. Tumor sizes were measured, and tumor volumes (mm^3^) were calculated as follows: length × width^2^ × 0.5. When tumors reached 100 to 200 mm^3^, 6 mice each were randomly allocated into groups (n = 6/group) administered osimertinib (40 µg per animal, 3 times per week, orally) or dasatinib (600 µg per mouse, 3 times per week, orally). Mice were anesthetized with injection of medetomidine (0.3 mg/kg), midazolam (4.0 mg/kg), and butorphanol (5.0 mg/kg) on day 15 (H1975) or day 22 (OR1) and tumors were collected. Tumor samples were stored at − 80 ℃.

### Patients and tumor samples

In this study, we analyzed 7 patients who were diagnosed with NSCLC at Kurume University hospital (Kurume, Japan). One patient (Case# 7) harboring EGFR exon 21 mutation received erlotinib. After erlotinib treatment, cancer relapsed with T790M mutation and then administration of osimertinib was followed for 1 year. Tumor tissues were obtained before and after osimertinib treatment. This study was approved by the Institutional Review Board of Kurume University Hospital (19146) and all patients provided informed consent in accordance with the Declaration of Helsinki.

### Immunohistochemical analysis

Paraffin-embedded samples of human lung cancer tissues were cut into 4-μm sections, mounted on coated glass slides, and incubated with anti-AXL antibody (1:100, #8661, Cell Signaling Technology), and anti-CDCP1 antibody (1:100, #4115, Cell Signaling Technology). Briefly, immunostaining with each antibody was performed on the same fully automated Bond-III system using onboard heat-induced antigen retrieval with epitope retrieval solution 2 (pH 9.0) for 30 min, and incubated with each antibody for 30 min. This automated system uses a Refine polymer detection system (Leica Microsystems, Newcastle, UK) with HRP (horseradish peroxidase)-polymer as secondary antibody and 3,3′ diaminobenzidine (DAB) as the chromogen. The slides were visualized using DAB.

## Supplementary Information

Supplementary Information.

## Data Availability

Microarray data generated and/or analyzed during the current study are available in the Gene Expression Omnibus (GEO) repository, Accession Number GSE201608. All other data are also included in this published article and its Supplementary Information files.
