# Supplementary material for: AXL/CDCP1/SRC axis confers acquired resistance to osimertinib in lung cancer
Source: Sci Rep. 2022 May 28;12:8983. doi: 10.1038/s41598-022-12995-8 (PMC9148303; doi:10.1038/s41598-022-12995-8)

Supplementary Information

AXL/CDCP1/SRC axis confers to acquired resistance to osimertinib in lung cancer

Yuichi Murakami[1,2], Daiki Kusakabe[3], Kosuke Watari[4], Akihiko Kawahara[5], Koichi Azuma[6], Jun Akiba[5], Masahiko Taniguchi[7], Michihiko Kuwano[1] and Mayumi Ono[1.8*]

Author affiliations
[1]Cancer Translational Research Center, St. Mary's Institute of Health Sciences, Kurume, Fukuoka, Japan
[2]Department of Pharmaceutics, Graduate School of Pharmaceutical Sciences, Kyushu University, Fukuoka, Japan
[3]Physical Chemistry for Life Science Laboratory, Faculty of Pharmaceutical Sciences, Kyushu University, Fukuoka, Japan.
[4]Department of Pharmacology, School of Medicine, University of California, San Diego, La Jolla, CA, USA
[5]Department of Diagnostic Pathology, Kurume University Hospital, Kurume, Fukuoka, Japan.
[6]Division of Respirology, Neurology and Rheumatology, Department of Internal Medicine, Kurume University School of Medicine, Kurume, Fukuoka, Japan
[7]Department of Surgery, St. Mary's Hospital, Kurume, Fukuoka, Japan
[8]Department of Pharmaceutical Oncology, Graduate School of Nursing, St. Mary's College, Kurume, Fukuoka, Japan

***Corresponding Author:**
Mayumi Ono, Ph.D.
Department of Pharmaceutical Oncology, Graduate School of Nursing, St. Mary's College
422 Tsubukuhonmachi, Kurume, Fukuoka, 830-8558, Japan;
TEL : +81-942-50-0721
FAX : +81-942-34-5926
E-mail: ono@st-mary.ac.jp

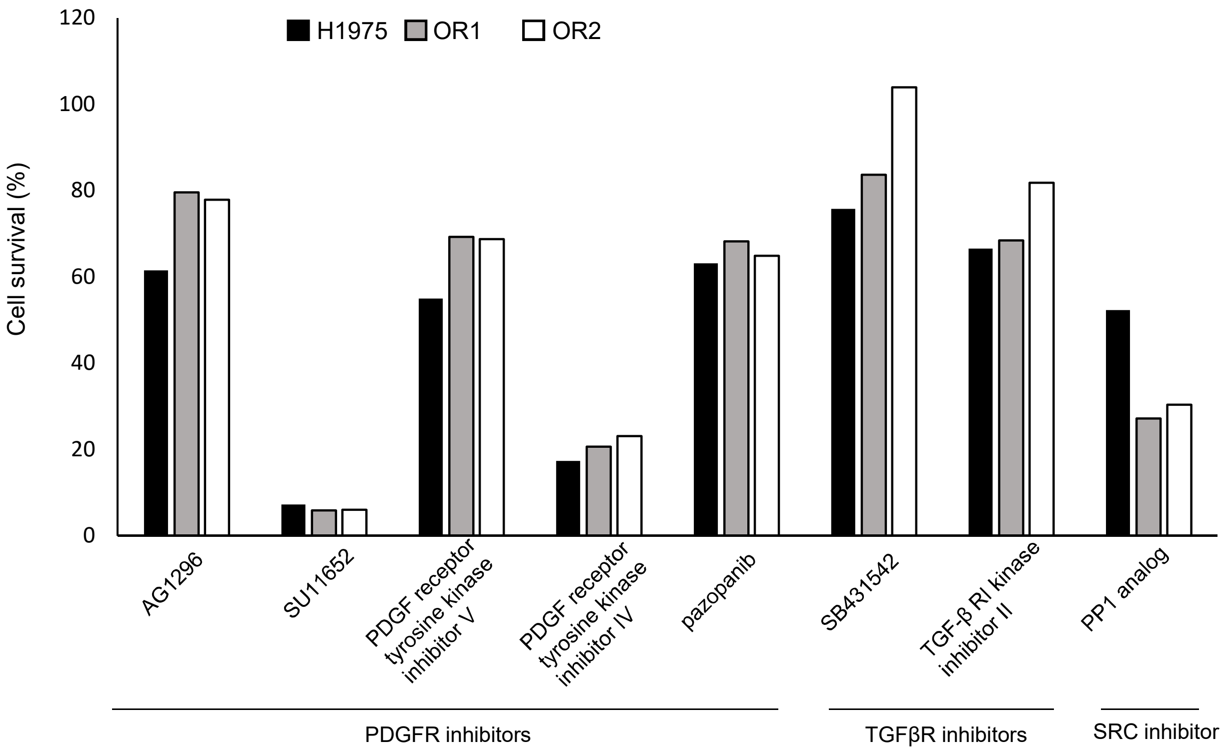

Figure S1. OR1 and OR2 cells could not exhibited high sensitivities to PDGFR and TGFβR inhibitors as compared with the parental cells. The sensitivity to kinase inhibitors was assessed using SCADS inhibitor kit. H1975, OR1, and OR2 cells were treated with kinase inhibitors at 5 μM for 96 hours and subjected to WST assay. Values are expressed as the percentage of the value in the absence of drugs.

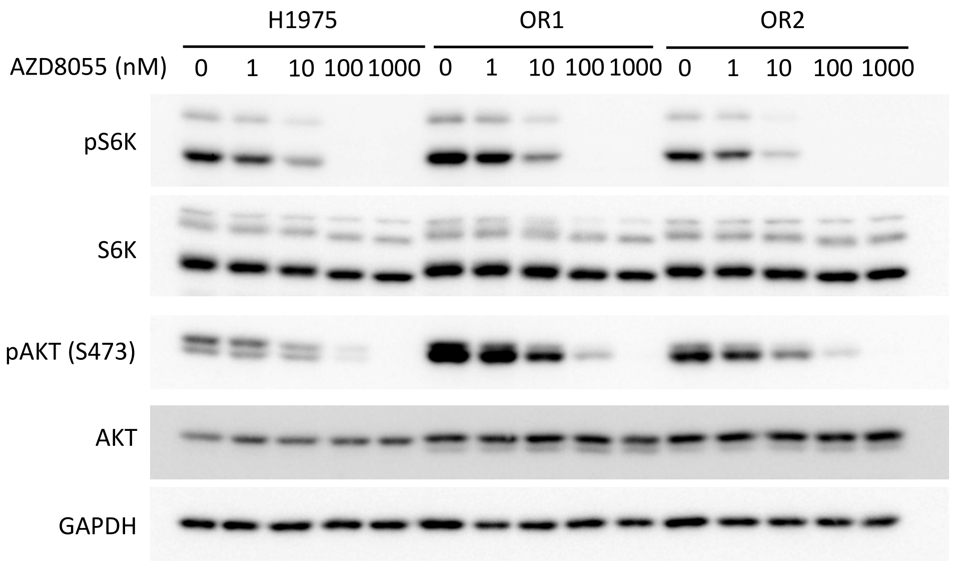

Figure S2. The inhibitory effects of AZD8055 on S6K and AKT activation. Cells were exposed to AZD8055 for 6 hours.

Table S1. Osimertinib resistant cells show collateral sensitivity to inhibitors of SFK

Drugs	Targets	IC50, μM[1]		
		H1975	OR1	OR2
Crizotinib	MET, ALK	1.82 (1)	1.38 (0.76)	1.20 (0.66)
PPP	IGF-1R	0.65 (1)	0.54 (0.83)	0.74 (1.14)
Sorafenib	RAF, VEGFR2 PDGFR	4.74 (1)	5.32 (1.12)	5.21 (1.10)
Dasatinib	SRC, ABL, KIT, EPH	0.23 (1)	0.0049 (0.021)	0.0207 (0.09)
PP1	SRC, LCK, FYN, KIT, EGFR	9.60 (1)	1.88 (0.20)	3.07 (0.32)
Saracatinib	SRC, LCK, FYN, KIT, EGFR	3.65 (1)	0.55 (0.15)	0.53 (0.15)
AZD8055	mTORC1, C2	0.048 (1)	0.015 (0.31)	0.016 (0.33)
GSK650394	SGK1/2	65.24 (1)	20.59 (0.32)	53.78 (0.82)
LY294002	PI3K	21.35 (1)	9.79 (0.46)	17.10 (0.80)
AKT inhibitor Ⅳ	AKT	0.19 (1)	0.21 (1.11)	0.28 (1.47)
U0126	MEK1/2	ND[2]	ND[2]	ND[2]
SP600125	JNK1/2, Aurora A	22.30 (1)	51.76 (2.32)	26.83 (1.20)

1) IC_{50} values (μM) are presented as average values of triplicate experiments. The relative drug sensitivity for each drug is presented in parenthesis as the IC50 of OR1/2 normalized to the IC_{50} of H1975 cells.
2) ND: not detectable, no cell toxicity is seen by the drug up to 30 μM

Original western blot images

Figure 1a

pEGFR

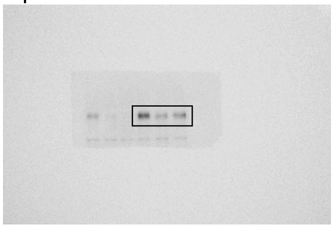

pEGFR marker

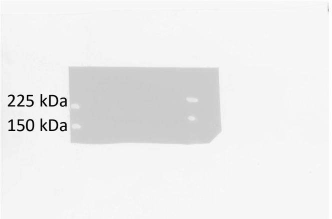

EGFR

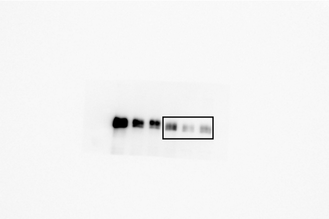

EGFR marker

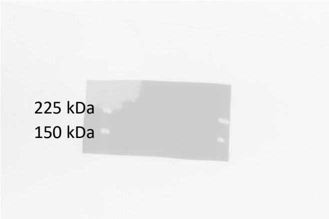

pHER2

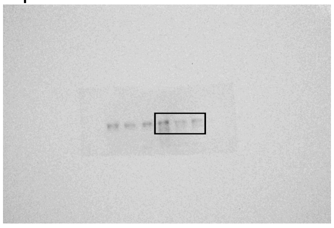

pHER2 marker

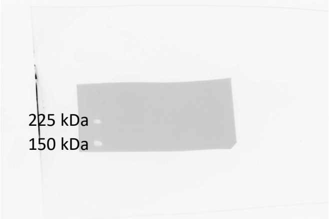

HER2

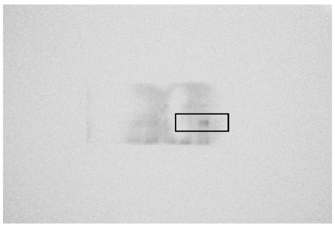

HER2 marker

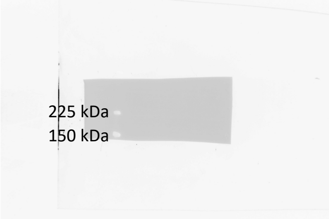

pHER3

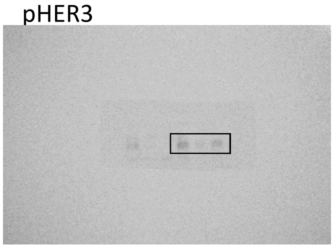

pHER3 marker

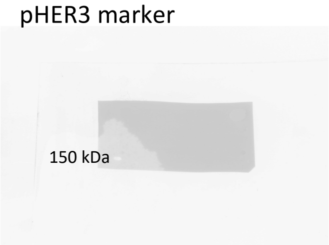

HER3

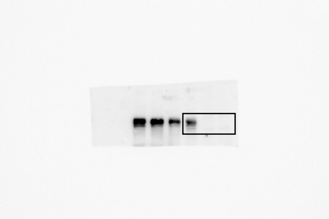

HER3 marker

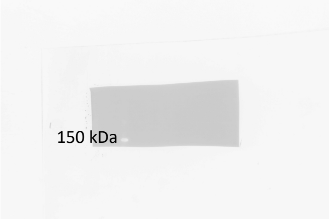

pMET

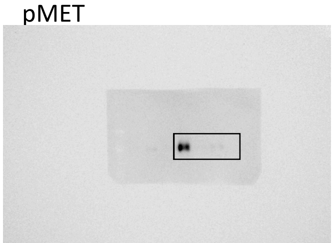

pMET marker

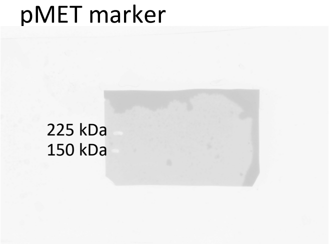

MET

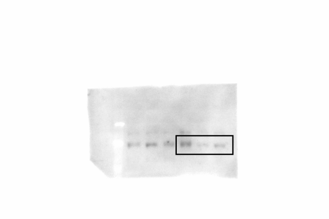

MET marker

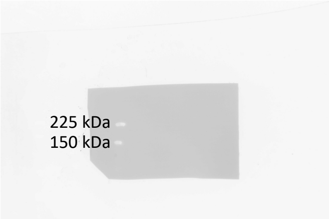

pSTAT3

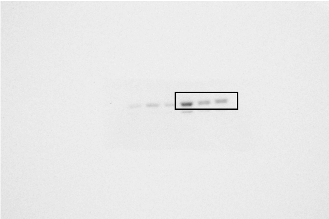

pSTAT3 marker

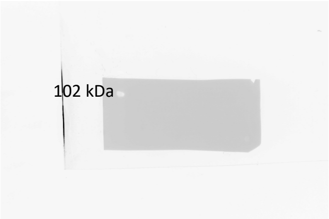

STAT3

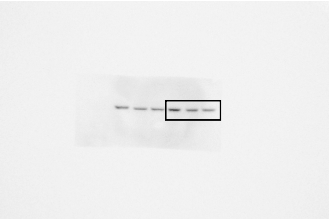

STAT3 marker

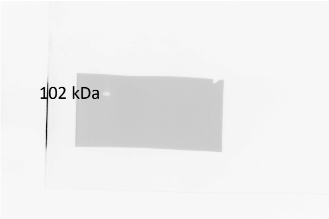

pAKT (T308)

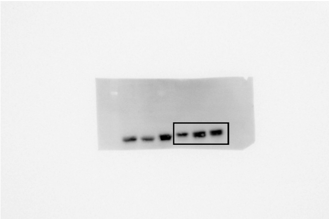

pAKT (T308) marker

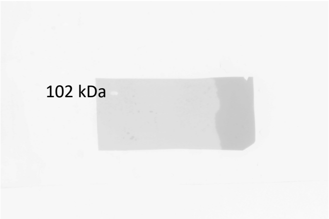

pAKT(S473)

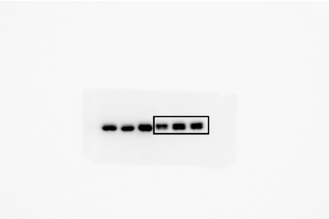

pAKT (S473) marker

AKT

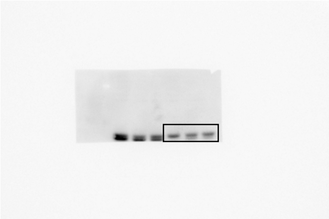

AKT marker

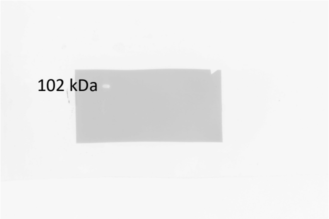

pPTEN

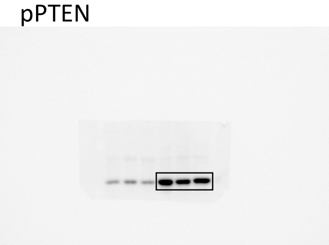

pPTEN marker

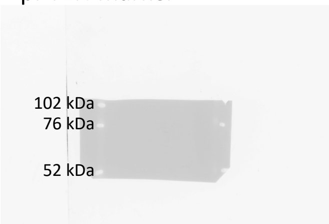

Figure 1a

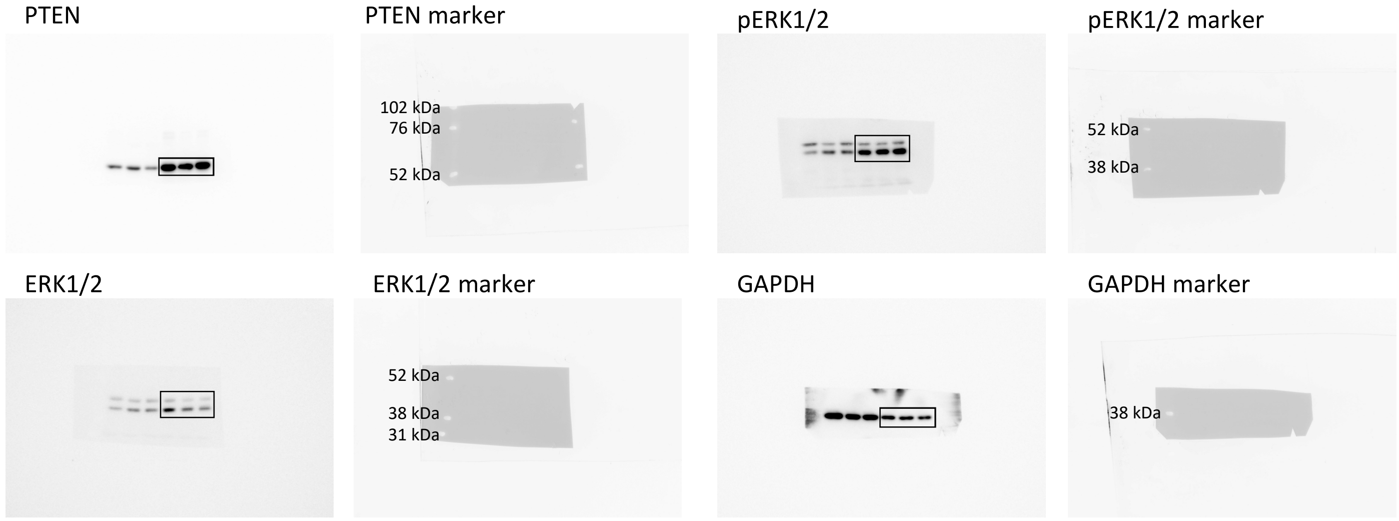
PTEN
PTEN marker
102 kDa
76 kDa
52 kDa
pERK1/2
pERK1/2 marker
52 kDa
38 kDa
ERK1/2
ERK1/2 marker
52 kDa
38 kDa
31 kDa
GAPDH
GAPDH marker
38 kDa

Figure 1b

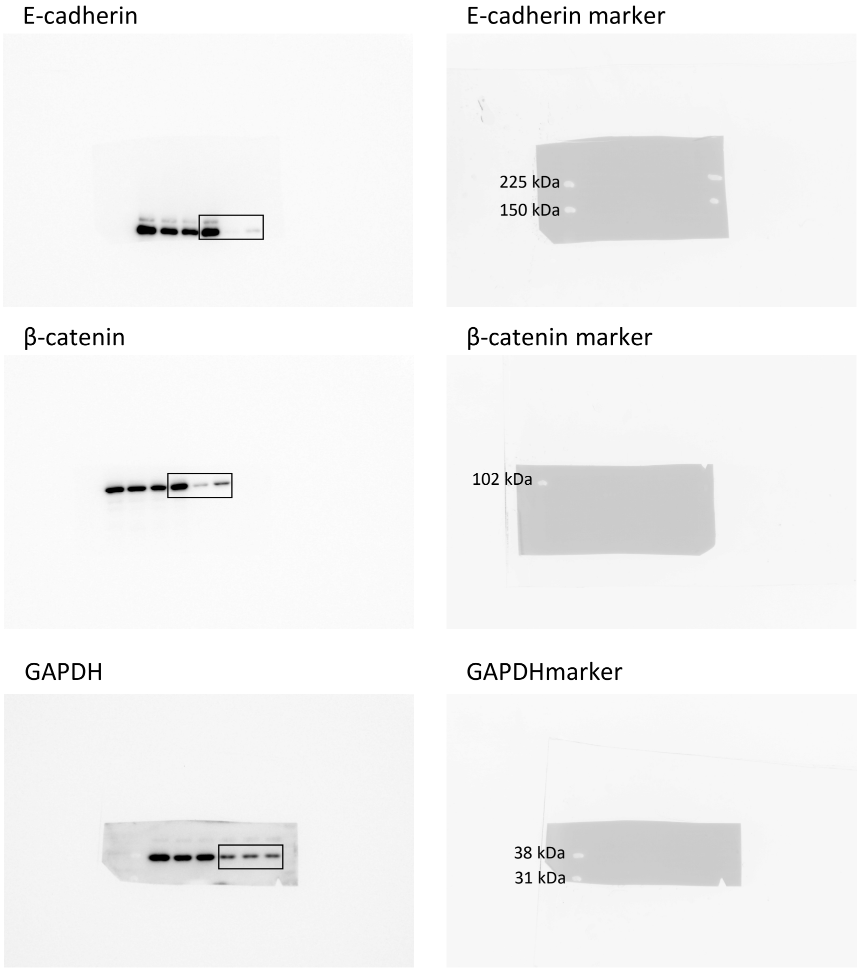
E-cadherin
E-cadherin marker
225 kDa
150 kDa
β-catenin
β-catenin marker
102 kDa
GAPDH
GAPDHmarker
38 kDa
31 kDa

Figure 1c

pEGFR

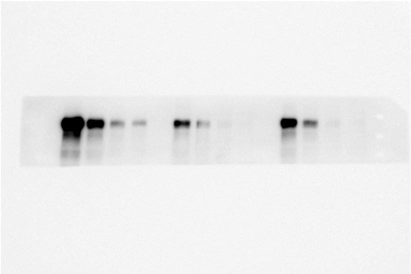

pEGFR marker

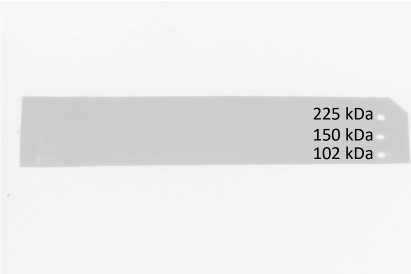

EGFR

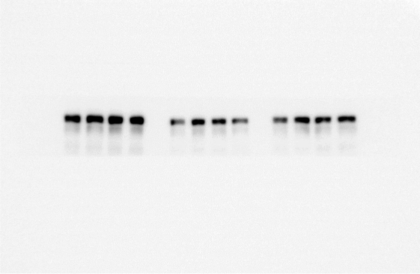

EGFR marker

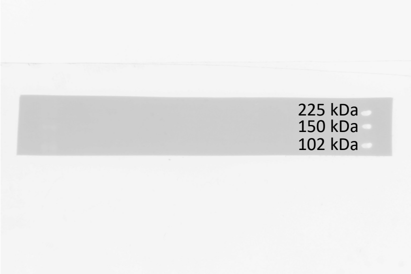

pAKT (T308)

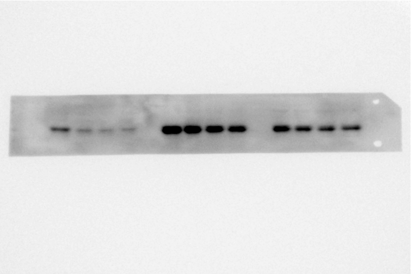

pAKT (T308) marker

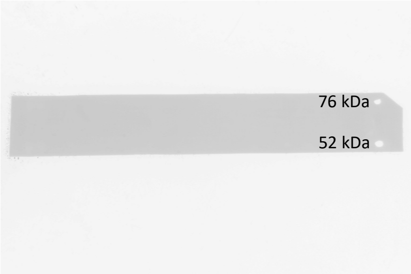

pAKT (S473)

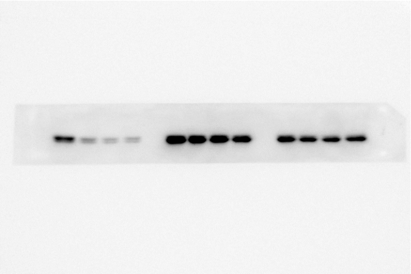

pAKT (S473) marker

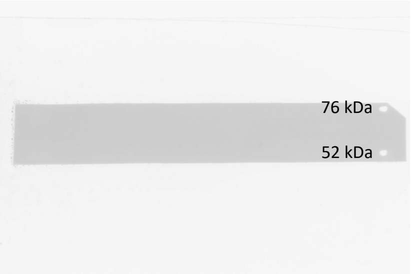

AKT

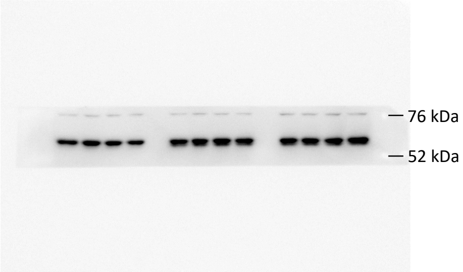

Figure 1c

pERK1/2

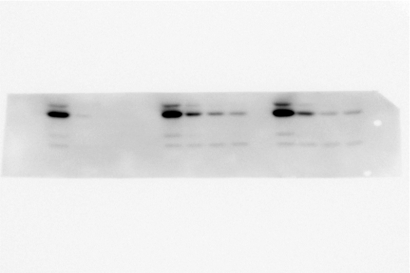

pERK1/2 marker

ERK1/2

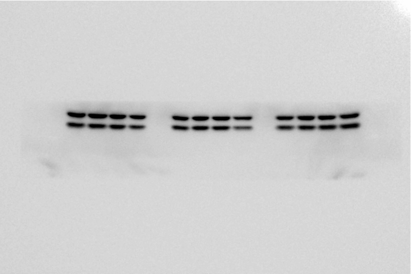

ERK1/2 marker

GAPDH

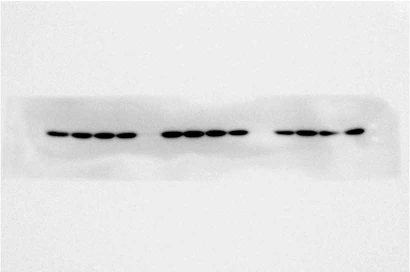

GAPDH marker

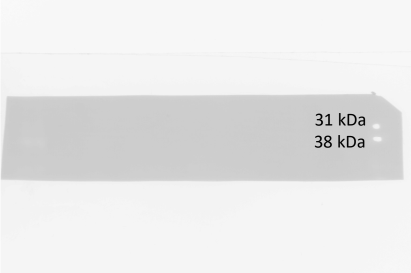

Figure 2a

AXL

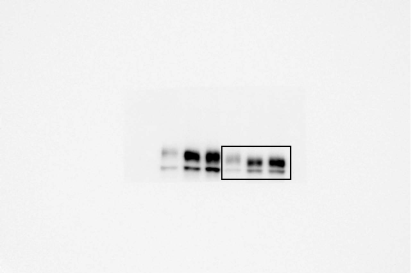

AXL marker

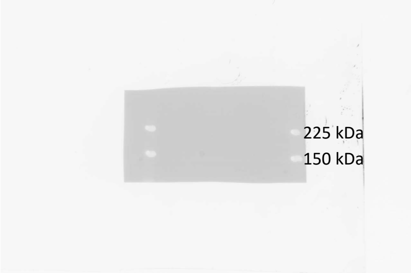

GAPDH

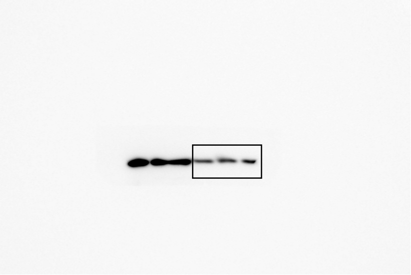

GAPDH marker

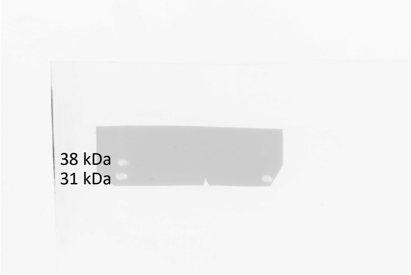

Figure 2b

AXL

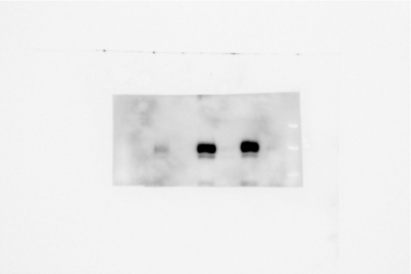

AXL marker

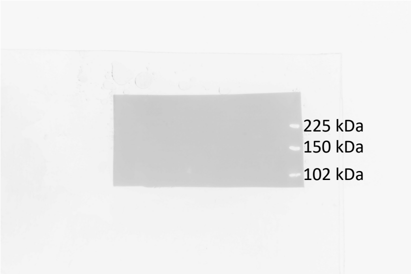

β-actin

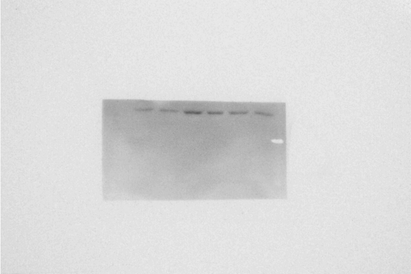

β-actin marker

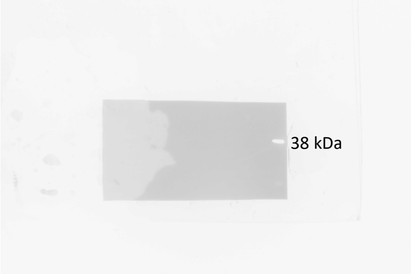

Figure 2d

AXL

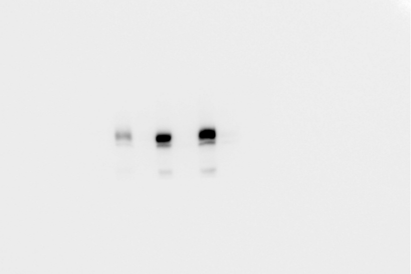

AXL marker

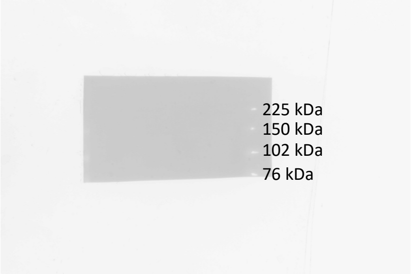

pEGFR (Y1068)

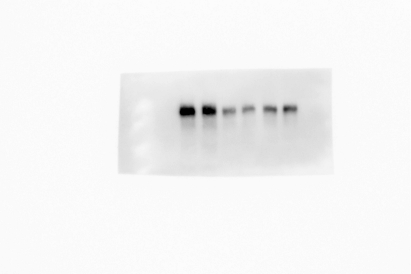

pEGFR (Y1068) marker

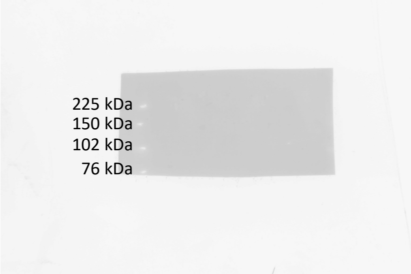

EGFR

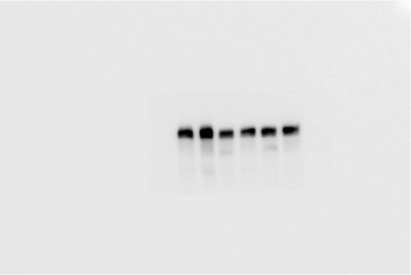

EGFR marker

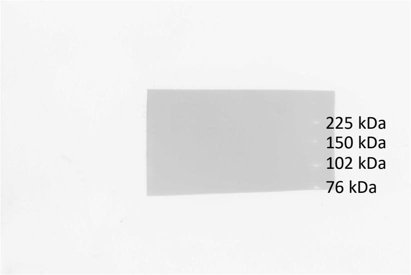

pSFK (Y416)

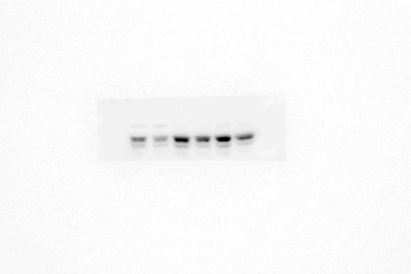

pSFK (Y416) marker

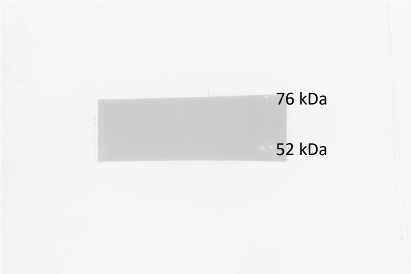

SRC

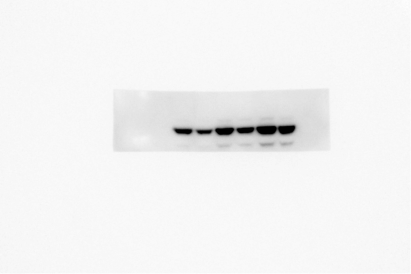

SRC marker

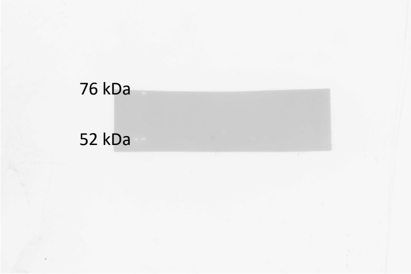

Figure 2d

pAKT (S473)

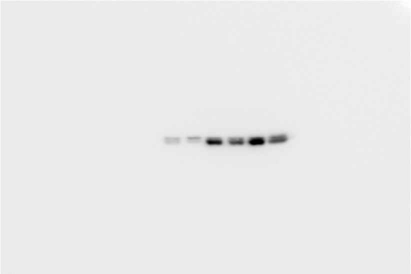

pAKT (S473) marker

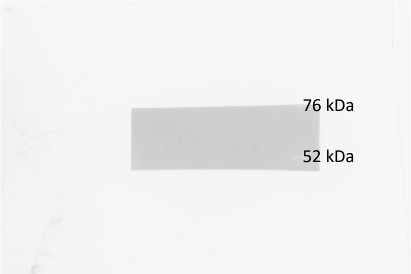

AKT

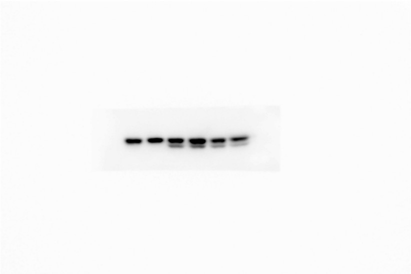

AKT

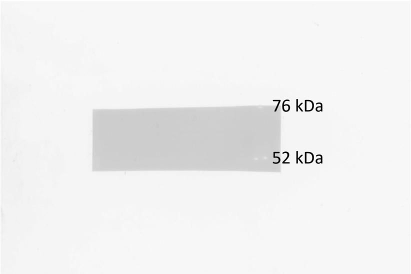

pERK1/2

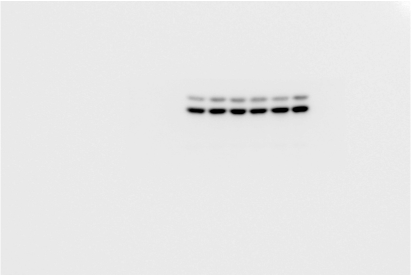

pERK1/2 marker

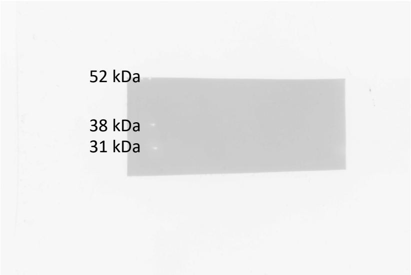

ERK1/2

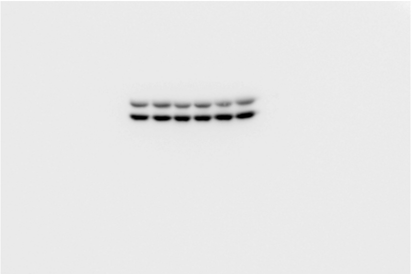

ERK1/2 marker

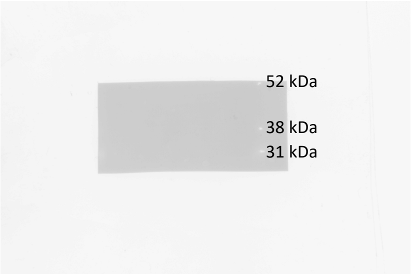

β-actin

β-actin marker

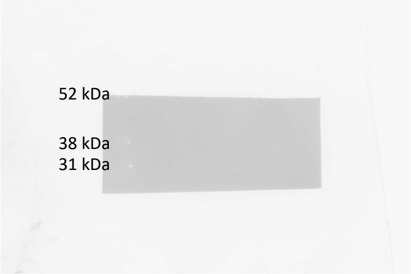

Figure 2f

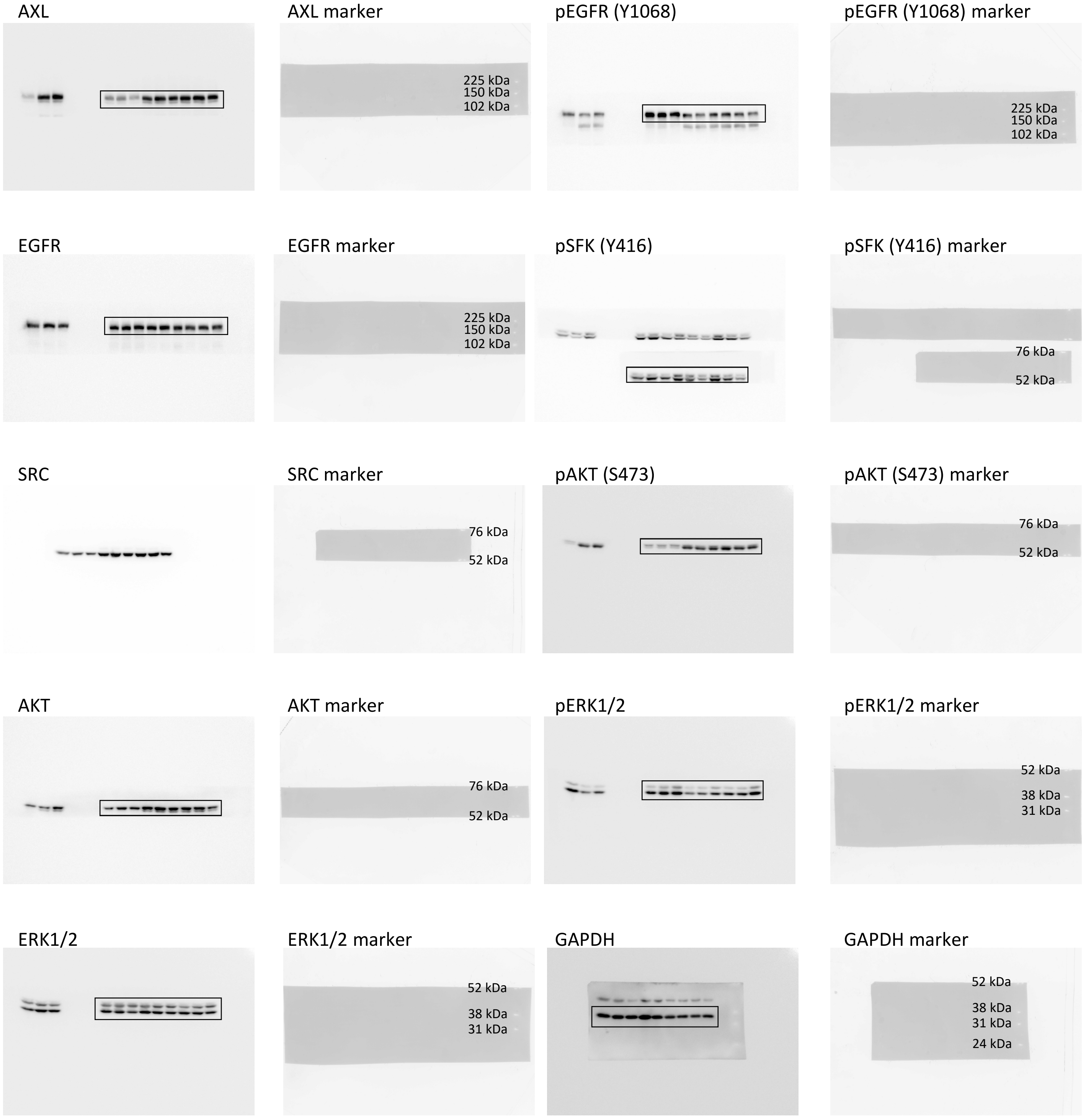
AXL
AXL marker
225 kDa
150 kDa
102 kDa
pEGFR (Y1068)
pEGFR (Y1068) marker
225 kDa
150 kDa
102 kDa
EGFR
EGFR marker
225 kDa
150 kDa
102 kDa
pSFK (Y416)
pSFK (Y416) marker
76 kDa
52 kDa
SRC
SRC marker
76 kDa
52 kDa
pAKT (S473)
pAKT (S473) marker
76 kDa
52 kDa
AKT
AKT marker
76 kDa
52 kDa
pERK1/2
pERK1/2 marker
52 kDa
38 kDa
31 kDa
ERK1/2
ERK1/2 marker
52 kDa
38 kDa
31 kDa
GAPDH
GAPDH marker
52 kDa
38 kDa
31 kDa
24 kDa

Figure 3a

CDCP1

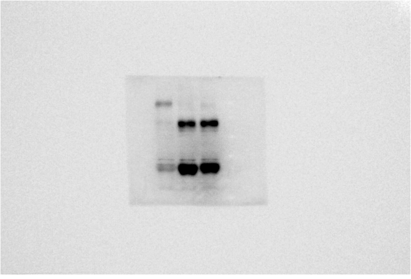

CDCP1 marker

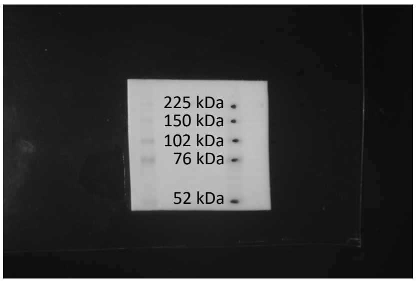

GAPDH

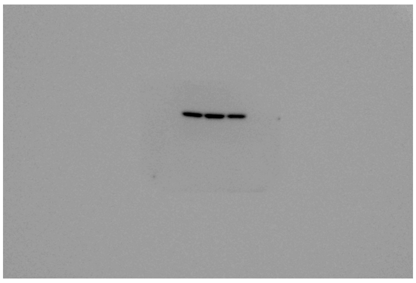

GAPDH marker

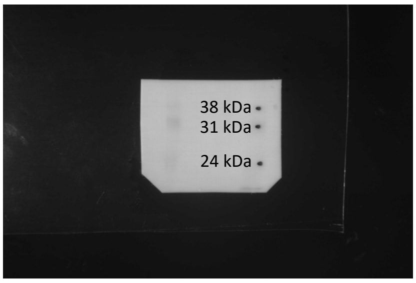

Figure 3b

pCDCP1

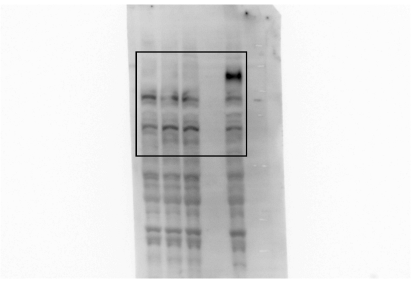

pCDCP1 marker

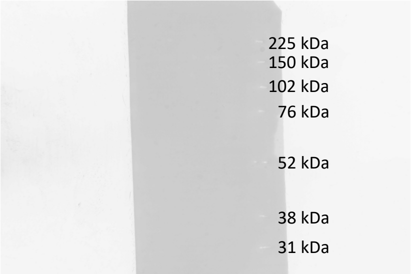

GAPDH

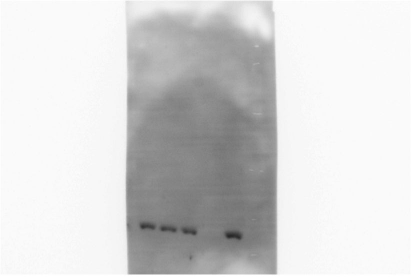

GAPDH marker

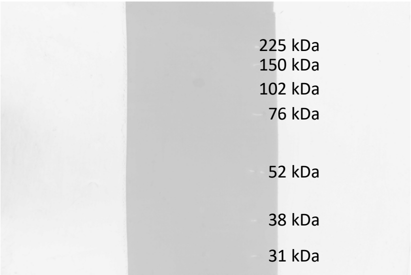

pCDCP1

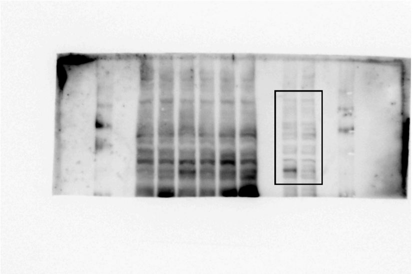

pCDCP1 marker

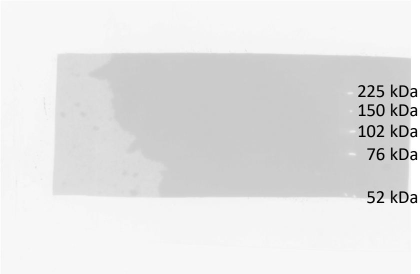

β-actin

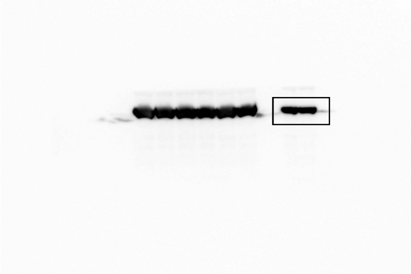

β-actin marker

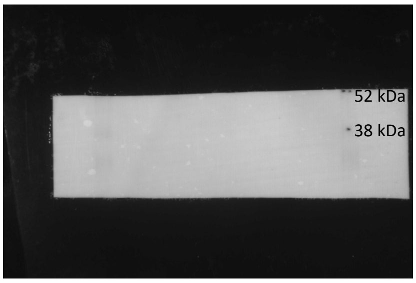

Figure 3d

CDCP1

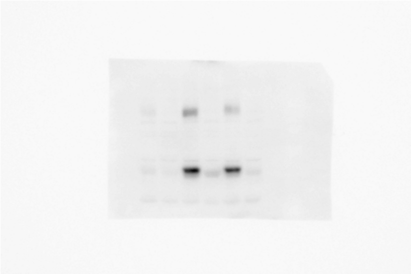

CDCP1 marker

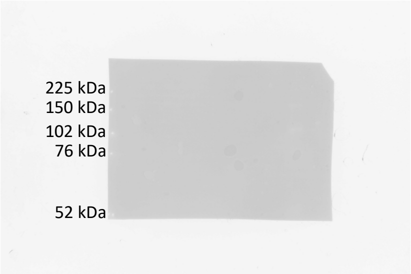

pSFK (Y416)

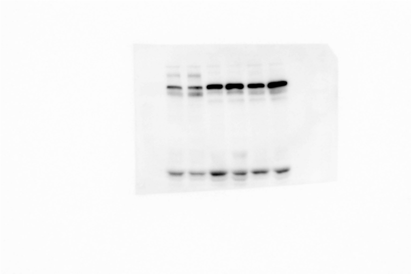

pSFK (Y416) marker

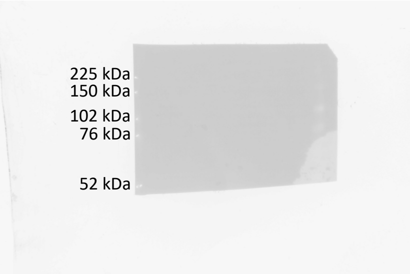

SRC

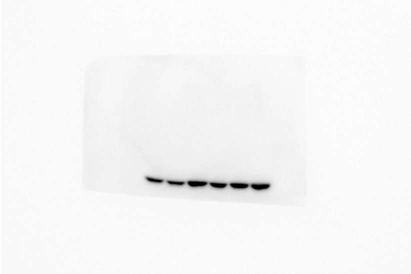

SRC marker

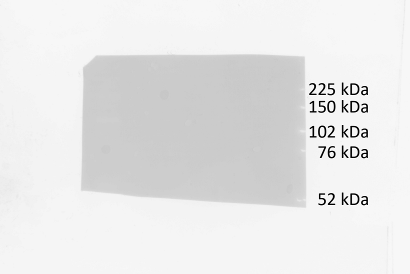

pAKT (T308)

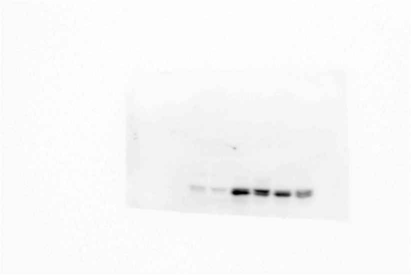

pAKT (T308) marker

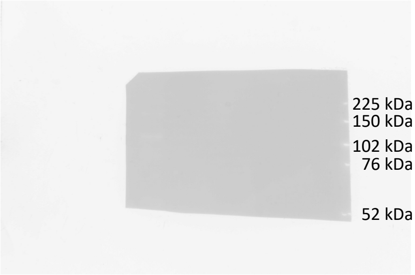

pAKT (S473)

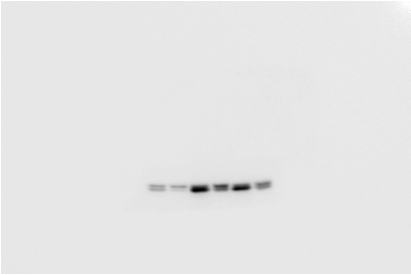

pAKT (S473) marker

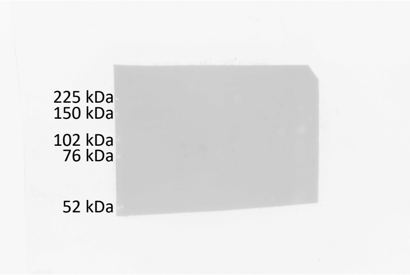

Figure 3d

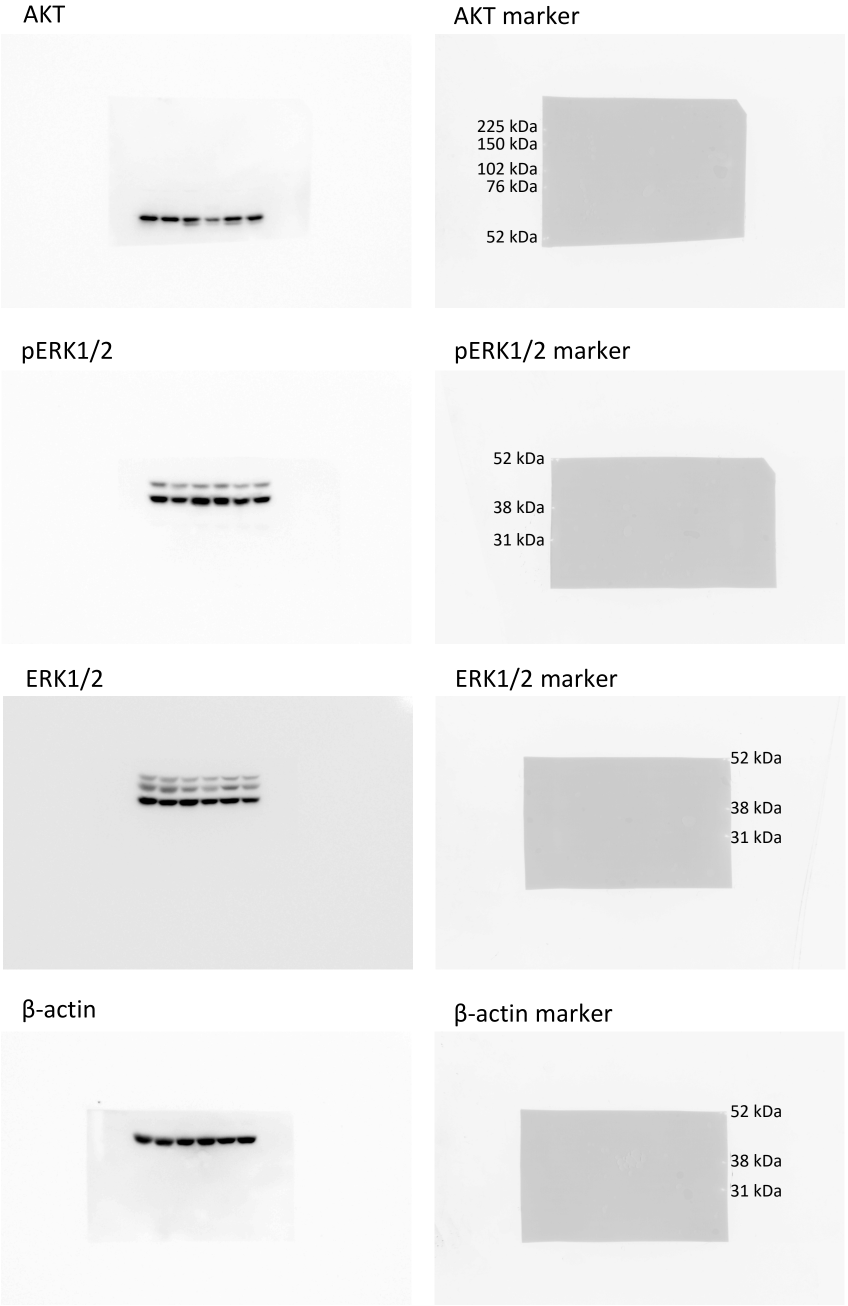
AKT
AKT marker
225 kDa
150 kDa
102 kDa
76 kDa
52 kDa
pERK1/2
pERK1/2 marker
52 kDa
38 kDa
31 kDa
ERK1/2
ERK1/2 marker
52 kDa
38 kDa
31 kDa
β-actin
β-actin marker
52 kDa
38 kDa
31 kDa

Figure 3e

SRC

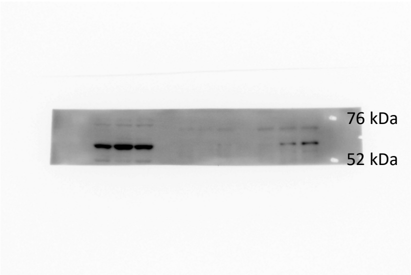

CDCP1

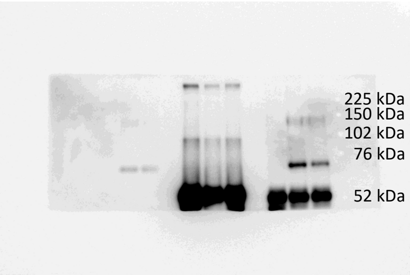

Figure 3f

CDCP1

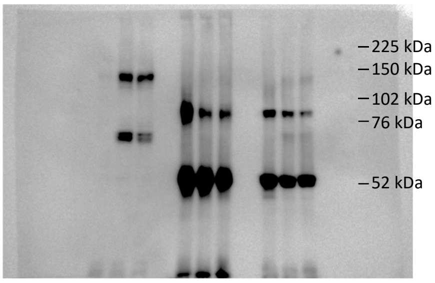

SRC

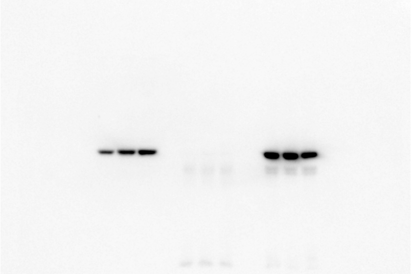

SRC marker

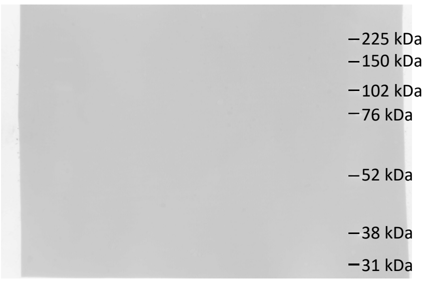

Figure 4a

SRC

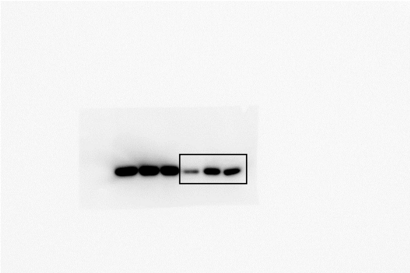

SRC marker

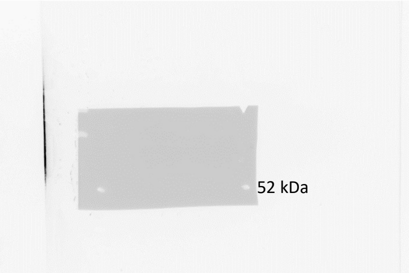

FYN

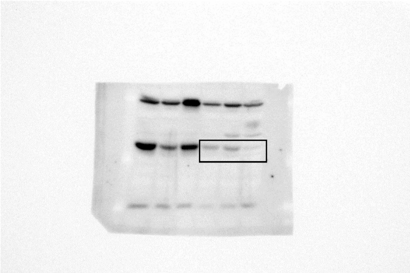

FYN marker

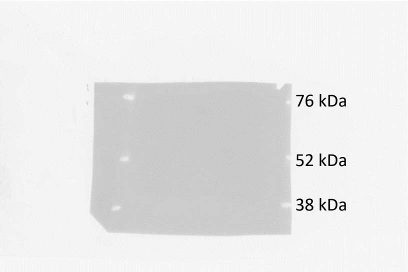

LYN

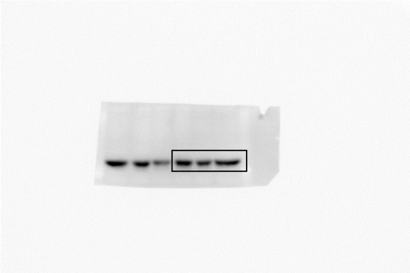

LYN marker

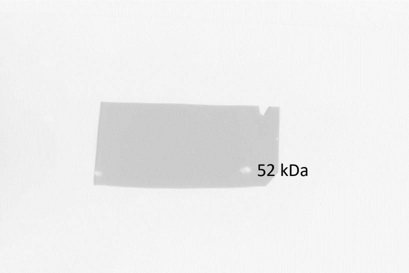

LCK

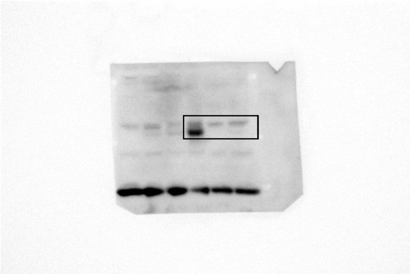

LCK marker

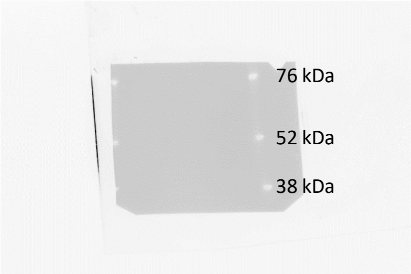

YES

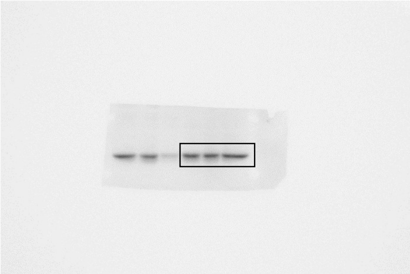

YES marker

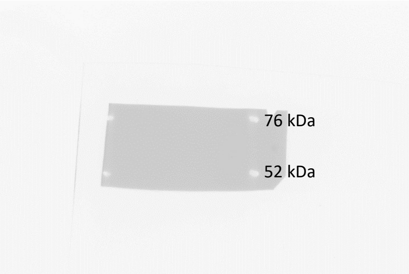

Figure 4a

CSK

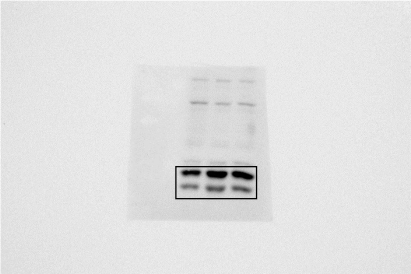

SRC marker

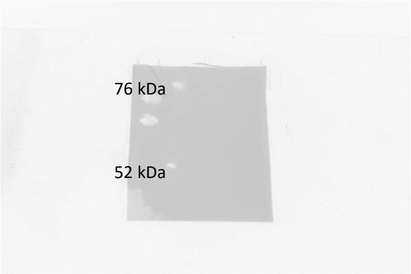

GAPDH

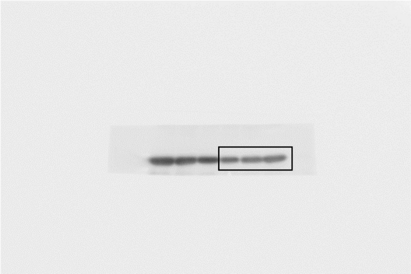

GAPDH marker

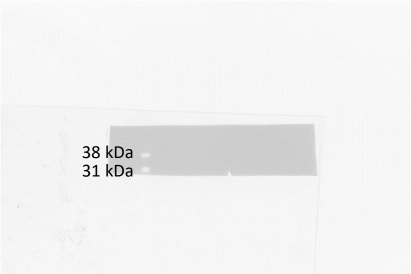

Figure 4c

pSFK

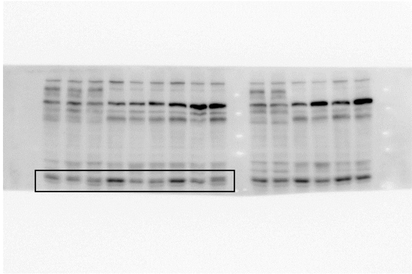

pSFK marker

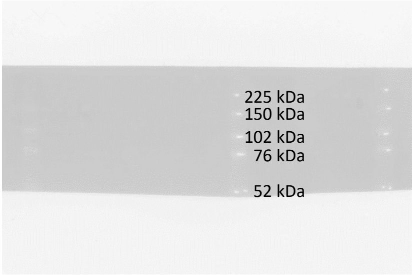

SRC

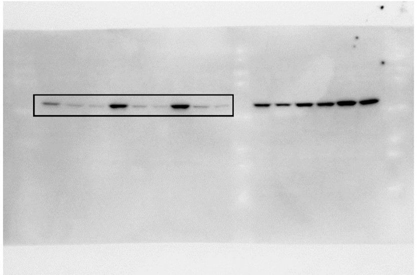

SRC marker

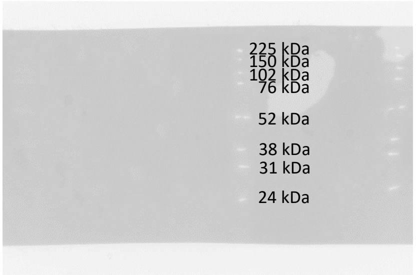

pAKT(T308)

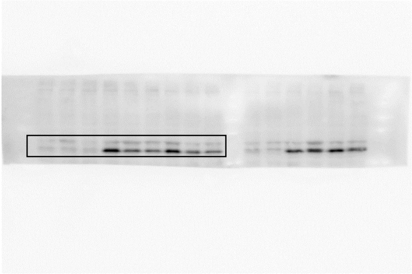

pAKT (T308) marker

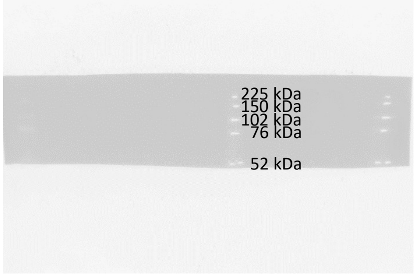

pAKT (S473)

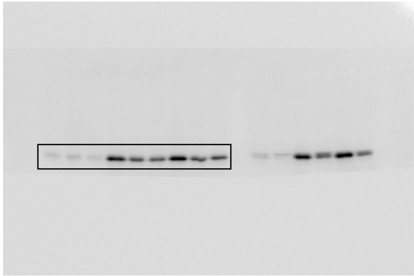

pAKT (S473) marker

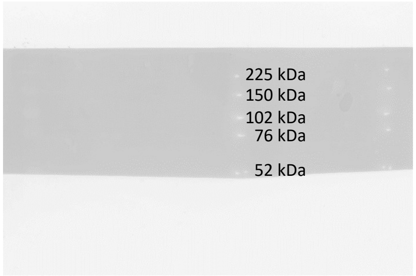

AKT

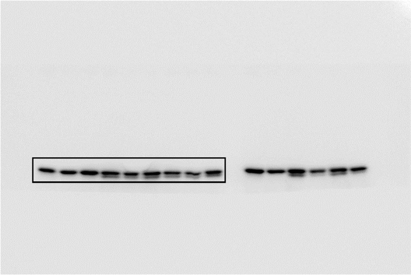

AKT marker

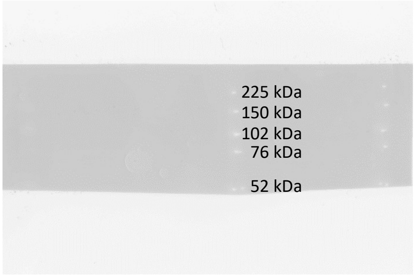

Figure 4c

pERK1/2

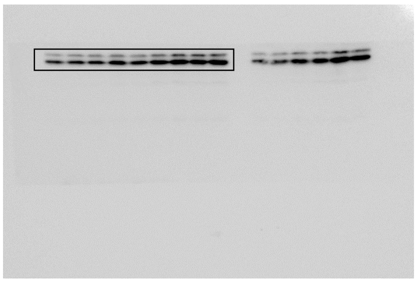

pERK1/2 marker

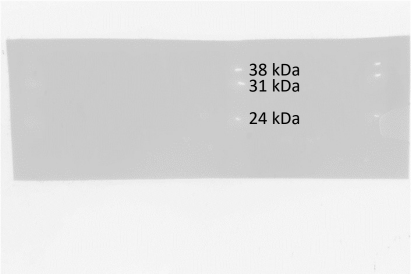

ERK1/2

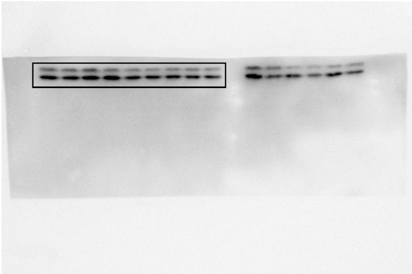

ERK1/2 marker

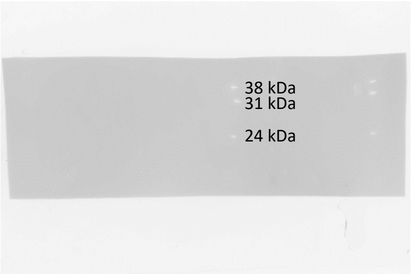

GAPDH

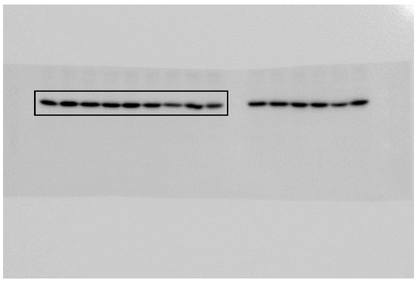

GAPDH marker

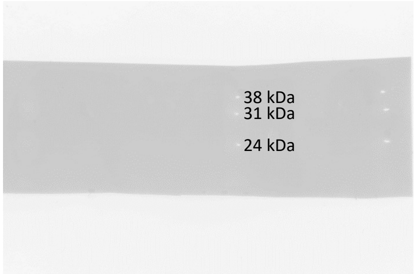

Figure 4e

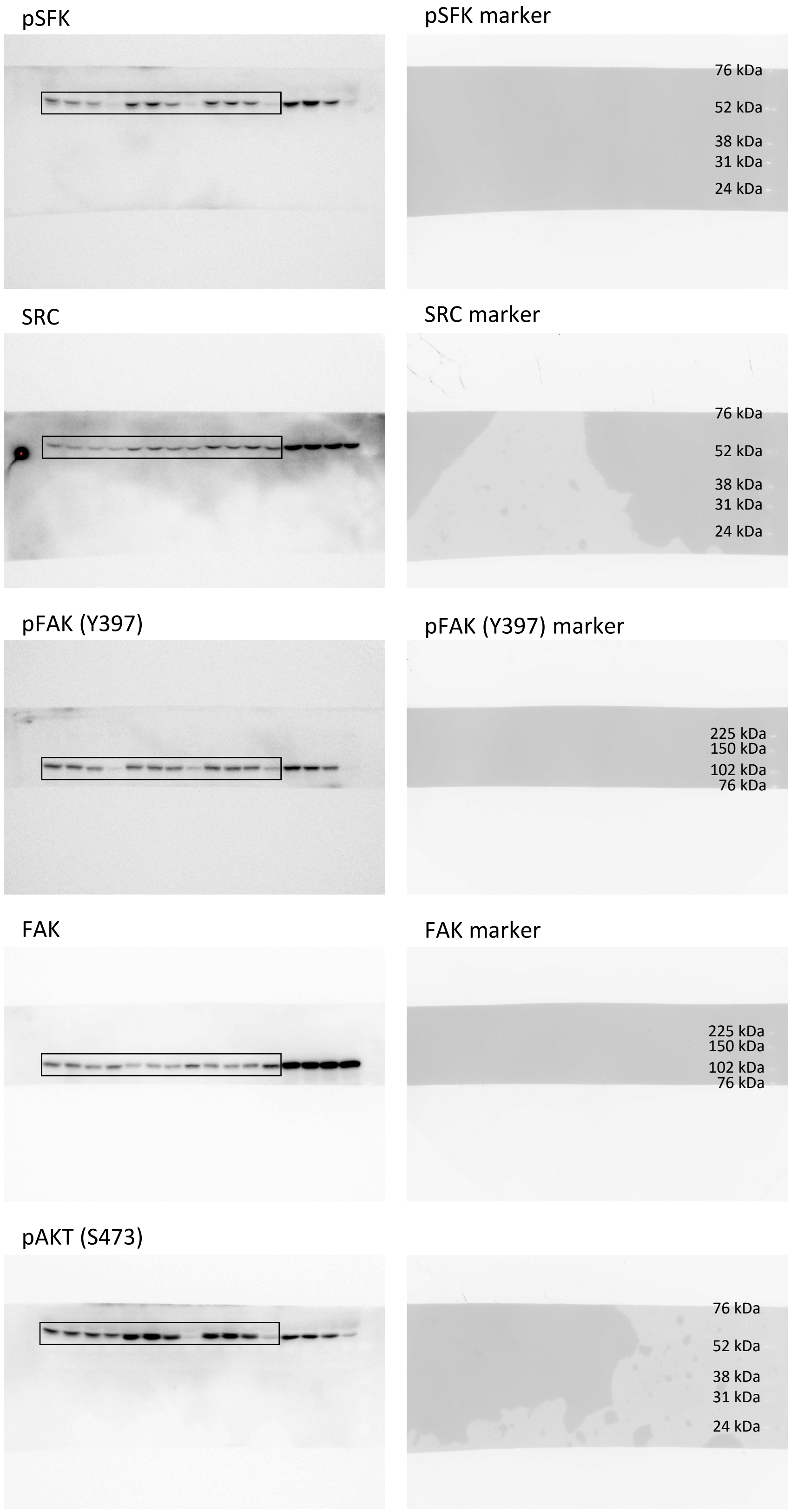
pSFK
pSFK marker
76 kDa
52 kDa
38 kDa
31 kDa
24 kDa
SRC
SRC marker
76 kDa
52 kDa
38 kDa
31 kDa
24 kDa
pFAK (Y397)
pFAK (Y397) marker
225 kDa
150 kDa
102 kDa
76 kDa
FAK
FAK marker
225 kDa
150 kDa
102 kDa
76 kDa
pAKT (S473)
76 kDa
52 kDa
38 kDa
31 kDa
24 kDa

Figure 4e

AKT

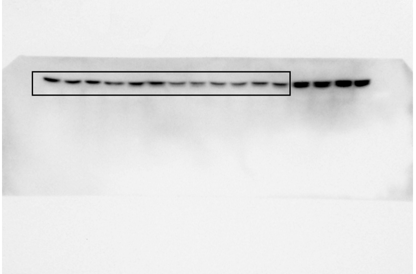

AKT marker

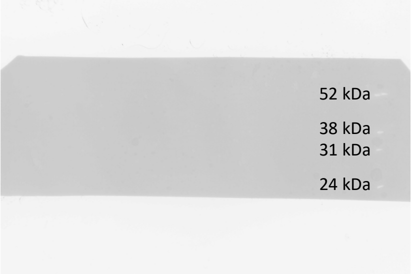

pERK1/2

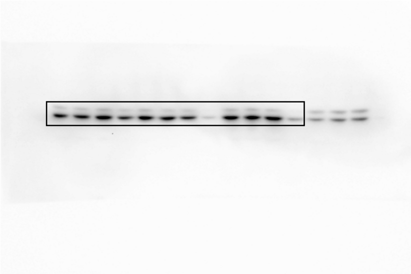

pERK1/2 marker

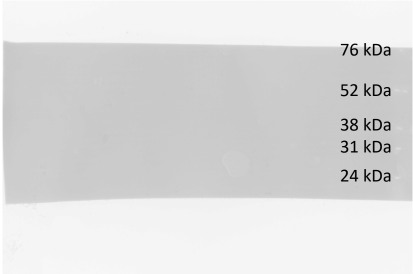

ERK1/2

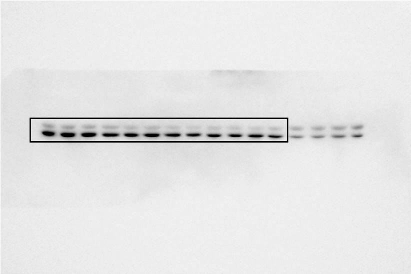

ERK1/2 marker

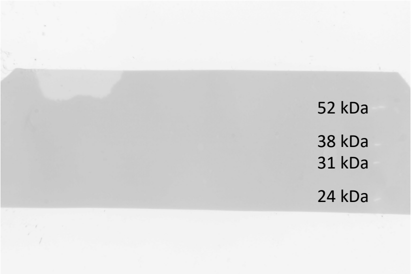

α-tubulin

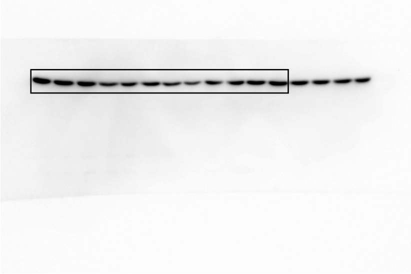

α-tubulin marker

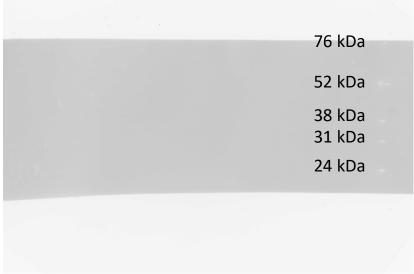

Figure 4g

pEGFR (Y1068)

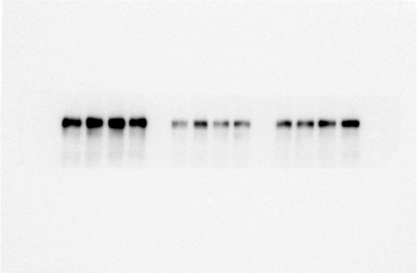

pAKT(S473) marker

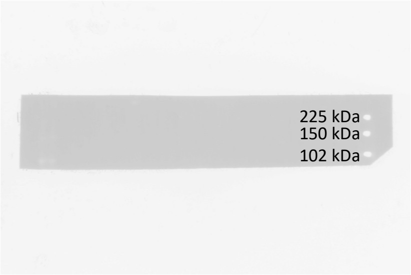

EGFR

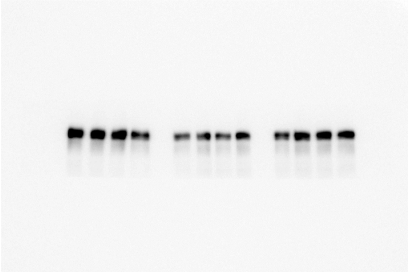

EGFR marker

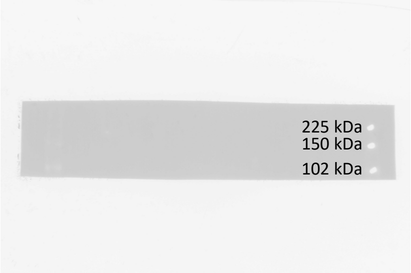

pSFK (Y416)

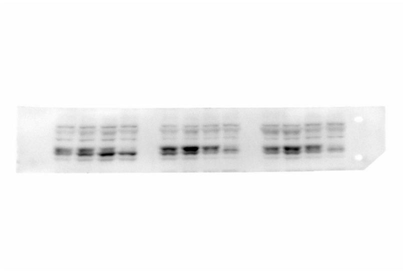

pSFK (Y416) marker

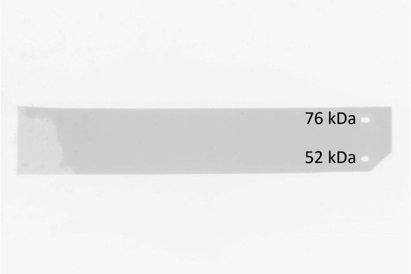

SRC

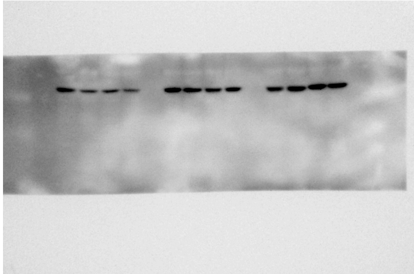

SRC marker

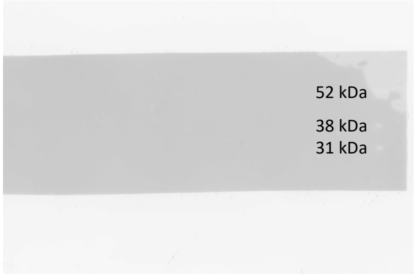

pAKT (T308)

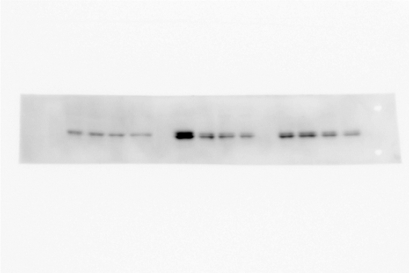

pAKT(T308) marker

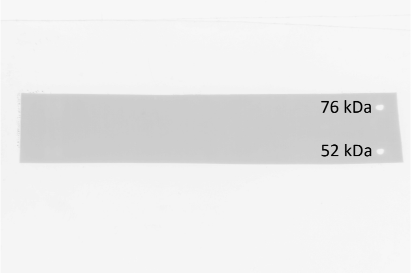

Figure 4g

pAKT (S473)

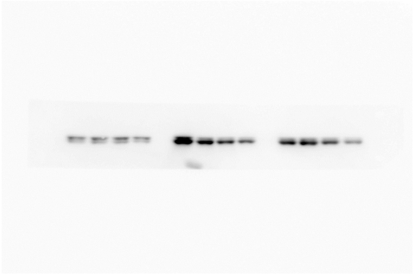

pAKT(S473) marker

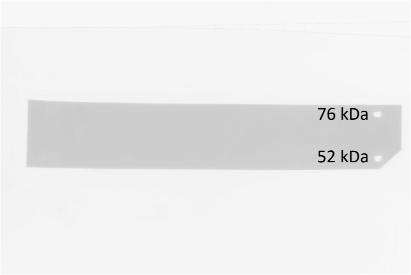

AKT

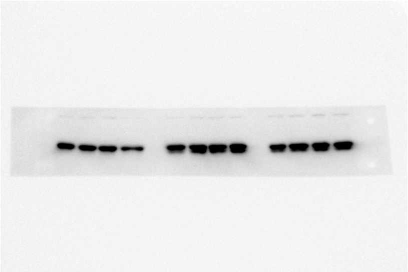

AKT marker

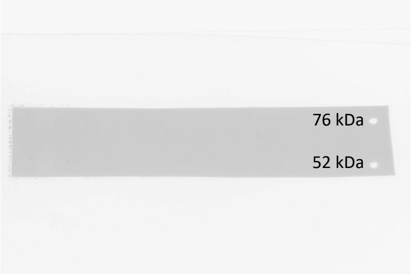

pERK1/2

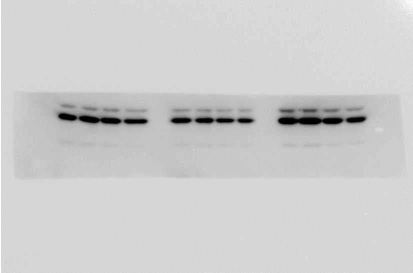

pERK1/2 marker

ERK1/2

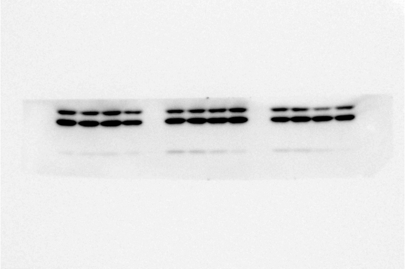

ERK1/2 marker

GAPDH

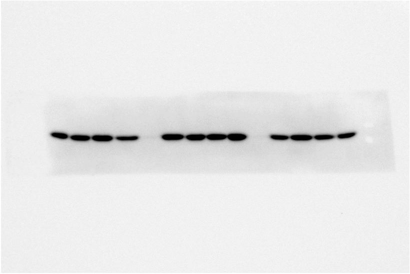

GAPDH marker

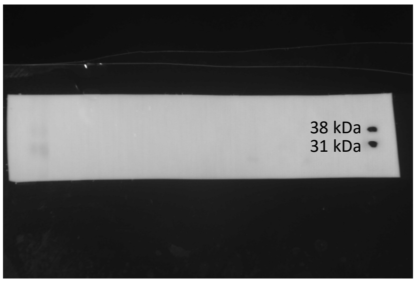

Figure 4i

EGFR

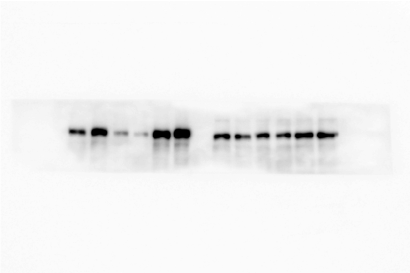

EGFR marker

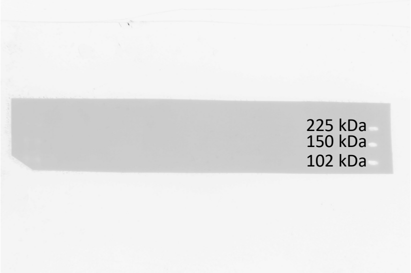

EGFR

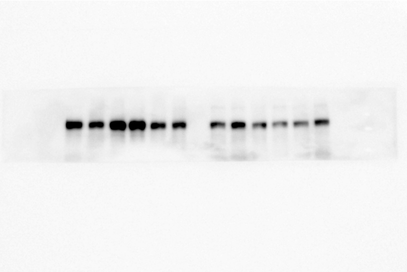

EGFR marker

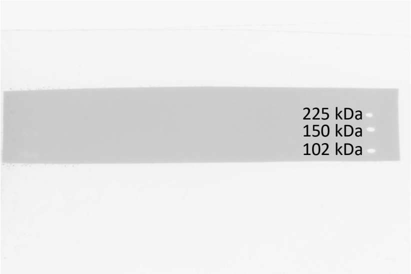

pSFK

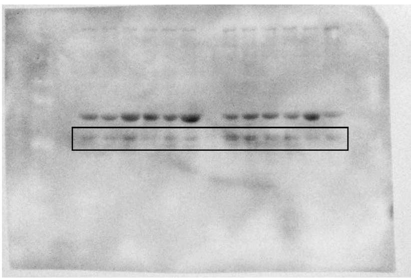

pSFK marker

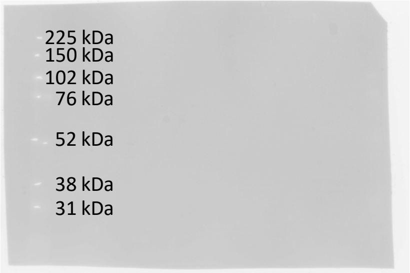

SRC

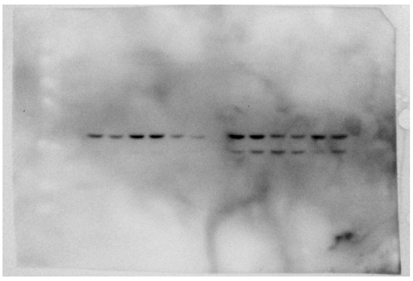

SRC marker

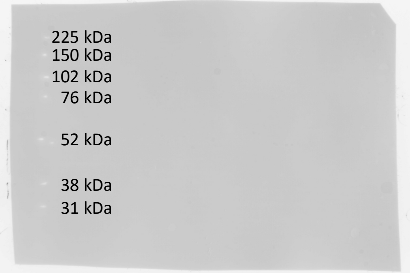

β-actin

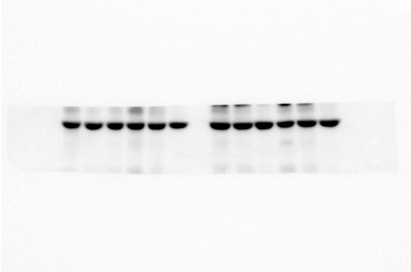

β-actin marker

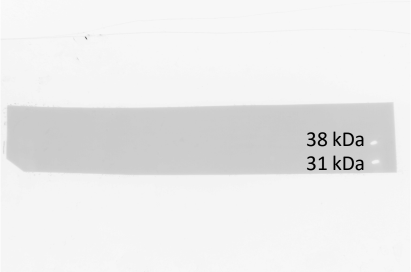

Figure 5c

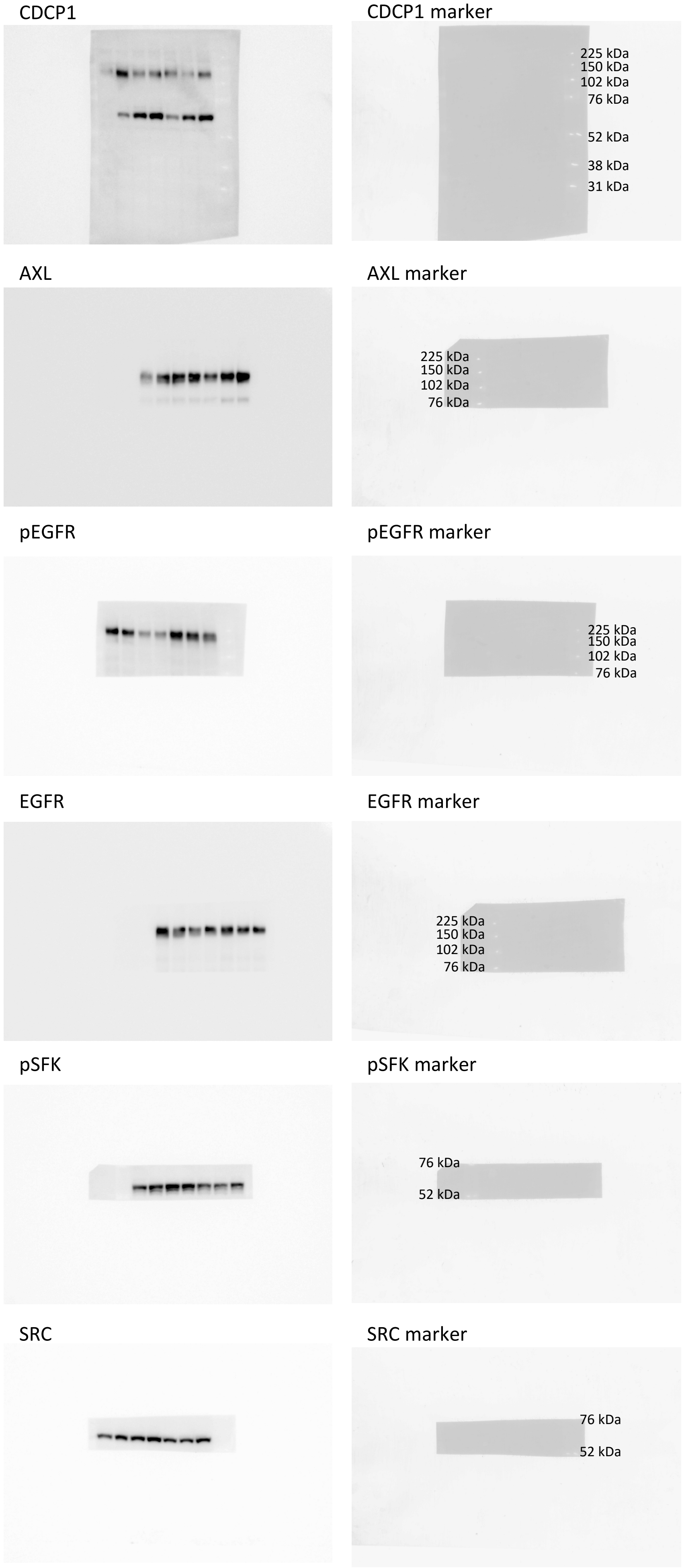

CDCP1
CDCP1 marker
225 kDa
150 kDa
102 kDa
76 kDa
52 kDa
38 kDa
31 kDa
AXL
AXL marker
225 kDa
150 kDa
102 kDa
76 kDa
pEGFR
pEGFR marker
225 kDa
150 kDa
102 kDa
76 kDa
EGFR
EGFR marker
225 kDa
150 kDa
102 kDa
76 kDa
pSFK
pSFK marker
76 kDa
52 kDa
SRC
SRC marker
76 kDa
52 kDa

Figure 5c

pAKT

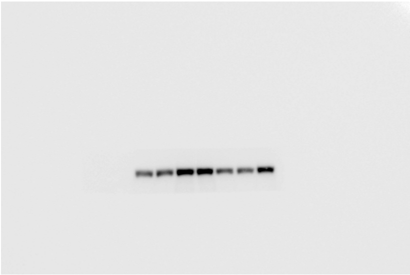

pAKT marker

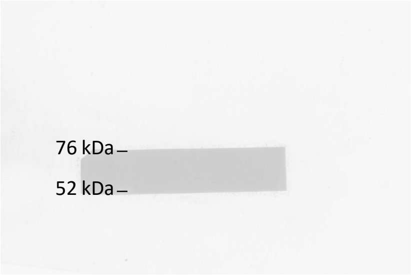

AKT

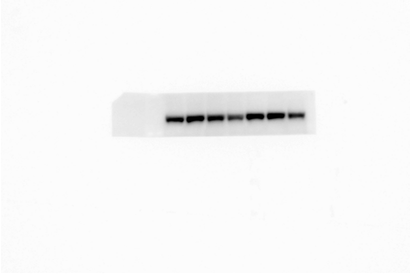

AKT marker

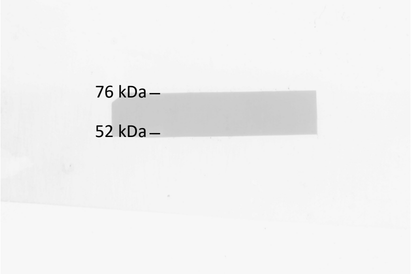

pERK1/2

pERK1/2 marker

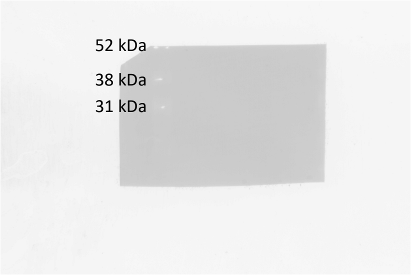

ERK1/2

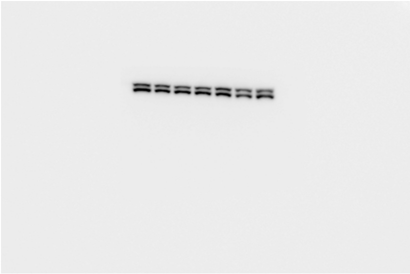

ERK1/2 marker

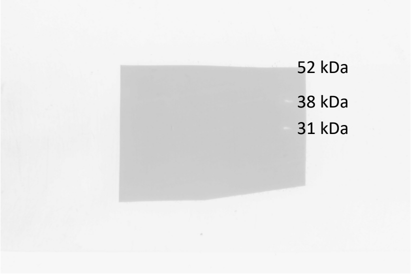

β-actin

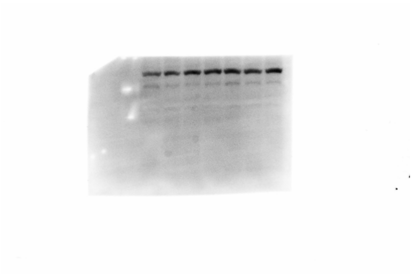

β-actin marker

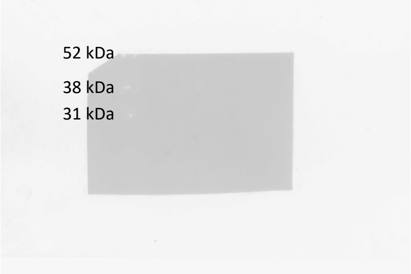

Figure S2

pS6K

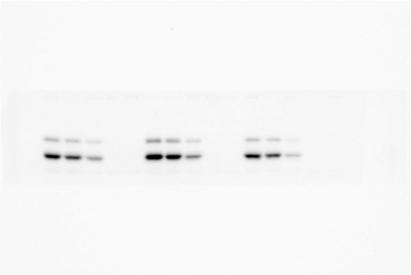

pS6K marker

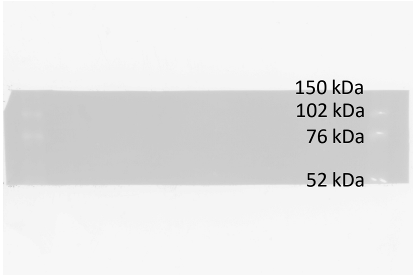

S6K

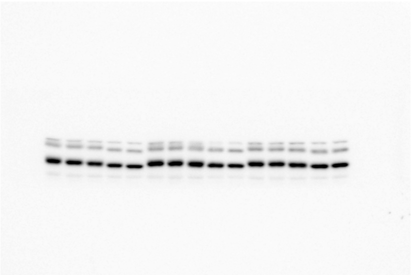

S6K marker

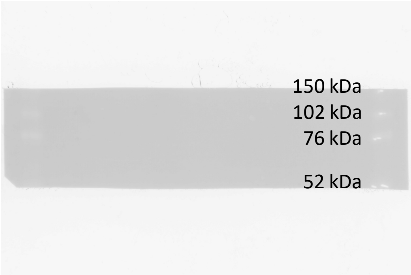

pAKT

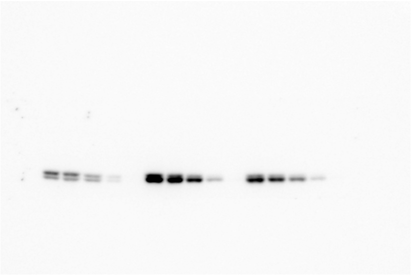

pAKT marker

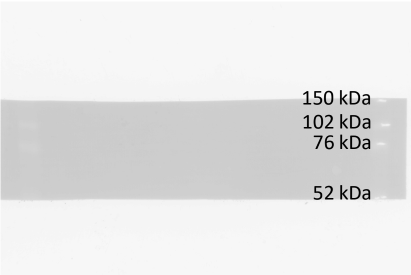

AKT

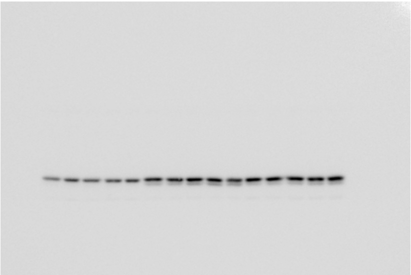

AKTmarker

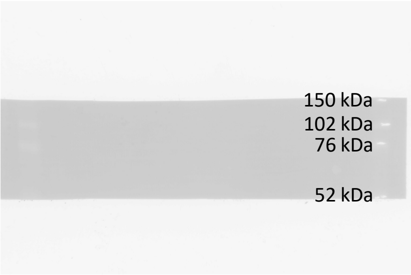

GAPDH

GAPDH marker

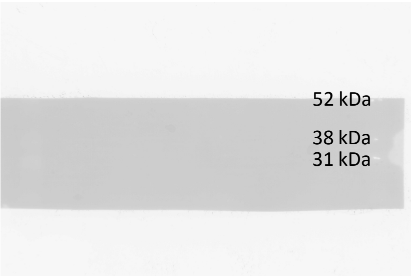

Supplement: Supplementary file 1 — Supplementary Information. [file 41598_2022_12995_MOESM1_ESM.pdf]
